# Dopamine D2 receptor modulating mPFC-BLA circuit contributes to chronic sleep deprivation-induced memory impairment in mice

**DOI:** 10.7150/thno.114797

**Published:** 2025-08-16

**Authors:** Jiaxuan Yang, Jiahui Sun, Zili Liu, Xia Tang, Yunyun Hu, Weida Shen, Yicheng Xie, Yue Jin, Haifeng Li, Xuekun Li, Yanjun Jiang, Matthew Tak Vai Chan, William Ka Kei Wu, Zhigang Liu, Xiaodong Liu, Yaoqin Hu, Jinpiao Zhu, Daqing Ma

**Affiliations:** 1 Perioperative and Systems Medicine Laboratory, Department of Rehabilitation, Department of Anesthesiology, Children's Hospital, Zhejiang University School of Medicine, National Clinical Research Center for Child Health, Hangzhou, 310015, China.; 2 Department of Anesthesiology, Zhongnan Hospital, Wuhan University, Wuhan, 430071, China.; 3 Shenzhen Institutes of Advanced Technology (SIAT), Chinese Academy of Sciences, Shenzhen, 518055, China.; 4 Key Laboratory of Novel Targets and Drug Study for Neural Repair of Zhejiang Province, School of Medicine, Hangzhou City University, Hangzhou, 310015, China.; 5 Binjiang Institute of Zhejiang University, Hangzhou, 310053, China.; 6 Department of Anaesthesia and Intensive Care, The Chinese University of Hong Kong, Prince of Wales Hospital, Hong Kong Special Administrative Region, China.; 7 Peter Hung Pain Research Institute, Li Ka Shing Institute of Health Sciences, The Chinese University of Hong Kong, Hong Kong Special Administrative Region, China.; 8 Division of Anesthetics, Pain Medicine & Intensive Care, Department of Surgery and Cancer, Faculty of Medicine, Imperial College London, Chelsea and Westminster Hospital, London SW10 9NH, United Kingdom.

**Keywords:** Chronic sleep deprivation, Memory impairment, Dopamine D2 receptor, Medial prefrontal cortex, Basolateral amygdala

## Abstract

**Background:** Chronic sleep deprivation (CSD) affects the orchestration of neural networks, leading to cognitive impairment, but the underlying molecular and neural circuitry mechanisms remain unknown.

**Methods:** Mice underwent a two-week CSD regimen, followed by spatial memory assessment using the Y-maze test and EEG gamma oscillation analysis. Dopamine D2 receptor (Drd2) expression in the medial prefrontal cortex (mPFC) was evaluated using transcriptomic and immunofluorescent analysis. The role of Drd2 in CSD-induced memory deficits was examined through local infusion of Drd2 agonists or antagonists into the mPFC. Neural circuit tracing, fiber photometry, and opto-chemogenetic approaches were used to assess Drd2 in the gating of the mPFC-basolateral amygdala (BLA) circuit-mediated memory impairment induced by CSD.

**Results:** CSD disinhibited dopaminergic input to the mPFC and impaired spatial memory in mice. A significant increase in Drd2 expression was found in the layers II/III of the mPFC after CSD. Infusion of Drd2 agonist into the mPFC induced memory deficits in naïve mice, while administration of the Drd2 antagonist reversed memory impairment caused by CSD. Drd2 was found to co-localize with Ca^2+^/calmodulin-dependent protein kinase IIα (CaMKIIα^+^) neurons in the mPFC that project to the basolateral amygdala (BLA). Activation of CaMKIIα^+^ neurons restored memory impairment induced by CSD through enhancing mPFC-to-BLA output and reversed memory defects induced by the Drd2 agonist.

**Conclusion:** Our findings demonstrated that excessive Drd2 signaling leads to cognitive impairment following CSD by suppressing mPFC-BLA neurotransmission, suggesting a possible therapeutic value of dopamine D2 receptor antagonists in relieving CSD-induced cognitive decline.

## Introduction

Sleep is essential for healthy cognition in a surprisingly diverse set of ways, including a healthy balance of gene transcriptome dynamics, synaptic plasticity, energy storage, and neural circuitry 1. Sleep is typically divided into two main states: rapid-eye movement (REM) and non-rapid-eye movement (NREM) sleep. NREM sleep is directly involved in memory consolidation and transfer 2, 3, but the role of REM sleep in this process has been a subject of debate. However, recent evidence suggested that neuronal activity occurring specifically during REM sleep is crucial for the consolidation of spatial and contextual memories 4. Using a combination of circuit-specific optical and single-cell electrophysiological recordings, previous studies indicated that REM sleep is necessary for social memory but not important for recognition memory 5. Sleep is crucial for synaptic plasticity during the processing of various types of memories. Sleep deprivation has prolonged effects on neuronal physiological functions during wakefulness, leading to impaired memory acquisition, consolidation, and recall in memory tasks 1, 6. Consequently, insufficient sleep can cause impaired memory, attention deficits, social dysfunction, anxiety, and even increased mortality 7-10. However, the underlying mechanisms driving these effects remain elusive and require further investigation.

The medial prefrontal cortex (mPFC) is a vital brain region in the memory network. By interacting with various other regions, mPFC supports executive functions, emotional regulation, and attention for comprehensive memory processing 11, 12. Indeed, mPFC is directly involved in memory encoding and retrieval, specifically in managing working, social, emotionally charged, and contextual memory through organizing and integrating information 13, 14. Moreover, mPFC is closely involved in memory processing during sleep 15, 16. For example, during REM sleep, the soma-to-dendritic decoupling occurred in Ca^2+^/calmodulin-dependent protein kinase II (CaMKII) positive pyramidal neurons in the mPFC; this process, in turn, promoted synaptic plasticity crucial for consolidation of associative learning 15. In contrast, chronic REM sleep deprivation in mice impaired the synaptic plasticity of CaMKII^+^ neurons, resulting in learning and memory defects 9. These studies highlight the mPFC as a brain region intricately involved in sleep disorders and their associated psychiatric and cognitive abnormalities. However, the specific molecular and neural circuitry of the mPFC linking to memory impairment induced by sleep deprivation is poorly understood.

In this study, we selectively interfere with REM sleep and investigate the molecular and neural circuitry changes affecting sleep architecture and memory processing in a mouse model of sleep deprivation.

## Methods

### Animals

All animal experiments were conducted in accordance with the ARRIVE Guidelines and approved by the Animal Welfare Committee of Zhejiang University, Zhejiang, China (ZJU20240600). 9-week-old male and female C57BL/6J mice (25 ± 5 g, obtained from Hangzhou Enlighten the Truth Laboratory Animal Technology Co., Ltd (Hangzhou, Zhejiang, China) were housed (5/cage) in a specific-pathogen-free room maintained at a temperature of 22 ± 2 °C and relative humidity between 50-60% with a 12-h light/dark cycle (light on at 8 am and light off at 8 pm). They had ad libitum access to food and water.

### Stereotaxic surgery

Mice were anesthetized with 2% isoflurane and placed on a digital stereotaxic instrument (68018, RWD Life Science, Shenzhen, China) for virus injection. After skull exposure, holes were made according to the following coordinates: for the mPFC, anterior-posterior (AP) +2.68 mm, medial-lateral (ML) +0.3 mm, dorsal-ventral (DV) -1.5 mm; and for the BLA, AP -1.25 mm, ML +3.0 mm, DV -4.75 mm. Micro-glass pipettes with an inner tip diameter of 9-10 μm, connected to a microsyringe pump (R-480, RWD Life Science, Shenzhen, China), loaded with AAV (see online Table S1), were injected at a rate of 20 nL/min for a total volume of 200 nL per injection. The pipette was left in the target brain region for 10 min to ensure optimal diffusion before being gradually withdrawn. Local anesthetic was applied locally before the incision was closed using nylon thread or sealed with dental cement in cases involving implantation. Afterwards, animals were placed on a warming pad for full recovery. They were kept for 21 days for further experimental use. Animals were excluded from experiments if the stereotaxic injection was missed in the target brain region.

### REM sleep deprivation

Mice with or without the above injection were subjected to REM sleep deprivation, which was induced using the modified multiple-platform technique as previously reported 9, 10, 17. Platforms with a diameter of 2.5 cm and a height of 5.0 cm were positioned in a water tank of the size 19 cm × 28 cm × 40 cm. These platforms were spaced 5.0 cm apart, and the tank was filled with water to a depth of approximately 2.0 cm below the top of the platforms. They were kept in the chamber for 20 h daily, from 2 pm to the next day at 10 am, and allowed to sleep naturally in their home cage for 4 h from 10 am to 2 pm over two weeks. The same tank with a glass panel was placed on the top of the platforms to allow mice (controls) to have normal sleep without falling into the water.

### EEG and EMG recording and sleep architecture analysis

Mice with or without the above injection were anesthetized with 2% isoflurane and placed on a digital stereotaxic instrument. Four wire electrodes (two placed bilaterally in the frontal skull; the other two placed bilaterally in the parietal skull) were screwed to record electroencephalograms (EEGs). An additional pair of insulated wire electrodes was inserted into the nuchal muscle for electromyogram (EMG) recording. The electrodes were connected to a micro-connector and fixed onto the skull surface with dental acrylic resin. After 7 days of postoperative recovery, the EEG and EMG signals were recorded at a sampling rate of 1000 Hz using the Medusa small animal electrophysiology recording system (Medusa, Bio-Signal Technologies, Nanjing, China). The sleep data were analyzed using a semi-automated sleep-scoring software with 10-s epochs, categorizing states into wake, REM, or NREM sleep, followed by manual correction, as we reported previously 10, 18.

### Gamma power analysis

Both Con and CSD mice underwent EEG recordings during the Y-maze test. Gamma oscillations (25-125 Hz) were bandpass filtered using Spike2 software (version 9.06; Cambridge Electronic Design Limited, UK). To measure changes in gamma activity associated with spatial memory processing, we specifically analyzed EEG signals during the 4-s periods before and after entry into the novel arm. Statistical analysis was performed using two-way repeated-measures ANOVA followed by Fisher's LSD post hoc test. Additionally, spectrograms were generated to compare gamma power dynamics across conditions qualitatively.

### *Ex vivo* brain slice electrophysiology recording

Whole-cell patch-clamp was employed to record inhibitory postsynaptic transmission in CaMKIIα^+^ neurons of the mPFC. One week after transfection with AAV2/9-CaMKIIα-mCherry, mice were subjected to the CSD protocol. Acute coronal brain slices were prepared in ice-cold modified artificial cerebrospinal fluid (ACSF) containing (in mM): 205 sucrose, 2.5 KCl, 1.25 NaH₂PO₄, 25 NaHCO₃, 10 glucose, 0.5 CaCl₂, and 7.5 MgCl₂. Slices were then incubated at 32-34 °C for 30 min in oxygenated standard ACSF composed of (in mM): 125 NaCl, 2.5 KCl, 1.3 NaH₂PO₄, 25 NaHCO₃, 25 glucose, 2 CaCl₂, and 1 MgCl₂. The mCherry^+^ neurons were targeted using glass pipettes (resistance: 3-5 MΩ) filled with CsCl-based solution optimized to enhance detection of inward inhibitory postsynaptic currents (IPSCs). Once a high-resistance seal (> 1 GΩ) was established, the membrane was ruptured to obtain a whole-cell configuration, and cells were voltage-clamped at -70 mV throughout the recording. To pharmacologically isolate inhibitory synaptic events and eliminate action potential-dependent activity, the external recording solution was continuously perfused with CNQX (10 µM), D-APV (50 µM), and TTX (1 µM), which block AMPA, NMDA receptors, and voltage-gated sodium channels, respectively. After a 5-minute stabilization period, miniature IPSCs (mIPSCs) were recorded for 3-5 min. Cells were excluded from analysis if they exhibited a change in membrane parameters > 15%, access resistance > 20 MΩ, or unstable baseline fluctuations. Both the frequency and amplitude of mIPSCs were quantified.

Three weeks after transfection of AAV-CaMKII-ChR2-EGFP in the mPFC of C57BL/6J mice. EGFP^+^ neurons were targeted for current-clamp recordings by glass pipettes filled with intracellular solution containing (in mM): 125 K-gluconate, 20 KCl, 0.5 EGTA, 10 HEPES, 10 Na₂-creatine, 4 Mg-ATP, and 0.3 Na-GTP. Action potentials were evoked by applying 2-s depolarizing current steps. Signals were acquired using a HEKA EPC-10 system. Series resistance (10-30 MΩ) was continuously monitored, and recordings were discarded if resistance changed by more than 30% during the experiment. For optogenetic stimulation, either a single 10-ms light pulse or a train of 1-ms pulses at 20 Hz for 1 s was delivered.

### Y-maze test

As reported previously 19, the Y-maze test was used to assess spatial learning and memory. During the training phase, one arm was chosen randomly as the novel arm, which was blocked with a barrier. Mice were allowed to explore the other two arms for 10 min. During the subsequent test phase, 1 h after training, the novel arm was made accessible, and mice were allowed to navigate all three arms for 5 min. The duration spent within the novel arm and the average speed were recorded and analyzed with the Any-maze video-tracking system (Stoelting Co., Wood Dale, Illinois, USA). The novel arm preference index was calculated as the ratio of time spent in the novel arm to the total trial duration.

### Micro-infusion

For cannula-guided micro-infusion of drugs, cannulas (Kedou Brain-Computer Technology Co. Ltd., Suzhou, China) were implanted into the mPFC (AP +2.68 mm; ML +0.3 mm; DV -1.5 mm) and fixed with dental cement; a dummy cannula was inserted to prevent block. Before injection, animals were allowed to recover for 1 week. Microinjections were performed using an injector cannula (outer diameter 0.25 mm) connected to a 10 µL micro-syringe (Gaoge, Shanghai, China) controlled by a micro-pump (UMP3, WPI, Worcester, MA, USA). Trifluoperazine 2HCl (TF) (0.1 mM), a Drd2 antagonist (Selleck Chemicals; Cat. S3201, Houston, Texas, USA), and quinpirole hydrochloride (QH) (2 mM), a Drd2 agonist (MedChemExpress; Cat. HY-B1752A, Monmouth Junction, New Jersey, USA) were dissolved in 0.9% saline and infused at 0.2 µL/min for 1 min, and their dose and action time were referred to previous work 20-22. If combined with the Y-maze test or sleep recording, micro-infusion was applied 10 min before the training phase of the test. The infusion sites were verified by imaging after sectioning and staining with DAPI. Mice with incorrect micro-infusion locations were excluded.

### Fiber-photometry

AAV2/9-hSyn-GRABeen_DA2m or AAV2/9-CaMKIIα-GCaMP7f was unilaterally injected into the mPFC for fiber-photometry recording. After injection, an optical fiber (230 μm in diameter, Xi'an Bogao Optoelectronic Technology Co., Ltd., Xian, China) was implanted, along with the securing of EEG/EMG electrodes. Mice were allowed to recover for 3 weeks and then placed in a soundproof chamber for 4-hour simultaneous recordings of dopamine fluctuations and EEG/EMG, from 2 pm to 6 pm. The signals of GRAB_DA_ were sampled at a rate of 40 Hz with a fiber photometry system (Thinker Tech Nanjing Co., Ltd., Nanjing, China). LED output intensity was adjusted to approximately 30 μW/mm^2^ to ensure optimal light excitation. Data were analyzed as we reported previously 23.

The GCaMP7f signal was recorded with the fiber-photometry system during stage 2 of the Y-maze test, along with a video recording. Data were selectively processed when mice entered the novel arm 2 s before and after the entry. The fluorescence change was presented as *ΔF/F* (*(F-F_0_)/F_0_*), where *F_0_* was the fluorescence signal averaged over a 2 s time window before the entry, and F was the peak fluorescence signal over a 2 s time window after the entry. *ΔF/F* values were shown with spectrograms or average plots, and a shaded area indicated the standard error of the mean (SEM).

### Chemogenetics

AAV2/9-CaMKIIα-hM3Dq or AAV2/9-CaMKIIα-hM4Di was injected into the mPFC, and then EEG/EMG electrodes were implanted. Mice were allowed to recover for 3 weeks. For CaMKIIα^+^ neuronal inactivation, 5 mg/kg Clozapine N-oxide (CNO, Shanghai Wanwuge Biotechnology Co., Shanghai, China) was injected i.p. 30 min before the Y-maze test. For CaMKIIα^+^ neuronal activation, mice underwent CSD after 7 days of viral injection, and 3 mg/kg CNO was injected i.p. 30 min before the Y-maze test on the second day following CSD. For sleep recording, 3 mg/kg CNO was injected i.p. 30 min before. To assess c-Fos expression, mice were euthanized 1 h after CNO administration.

### Neural tracing

AAV2/9-CaMKIIα-EGFP was transduced into mPFC CaMKIIα^+^ neurons. Mice were allowed to recover for 21 days and euthanized for immunohistochemistry. Sequential coronal sections of brains were acquired, and fluorescent images were captured using Pannoramic SCAN (3DHISTECH Ltd., Budapest, Hungary) and registered to the brain atlas (Mouse Brain in Stereotaxic Coordinates, second edition). Images were further processed with SlideViewer (v2.6, 3DHISTECH Ltd.) and Adobe Photoshop (v2020-21.0.0, Adobe Systems).

### Optogenetics

AAV2/9-CaMKIIα-hChR2 was injected unilaterally into the mPFC to activate CaMKIIα^+^ neuronal somas and their axon terminals in the BLA. Optical fibers were implanted ipsilaterally in either the mPFC or BLA. Mice were allowed to recover for 7 days and subjected to CSD. Neuronal activation was induced by blue light (473 nm, 4-5 mW, 20-ms pulses, 10 Hz; Thinker Tech Nanjing Co., Ltd., Nanjing, China) in 5 cycles of 1 min bursts followed by 1 min intervals during stage 1 of the Y-maze test. Stage 2 was conducted without light stimulation 1 h after stage 1. Mice were then euthanized for c-Fos staining 1 h after a 10 min duration of light stimulation.

AAV2/9-CaMKIIα-eNpHR was injected bilaterally into the mPFC to inactivate CaMKIIα^+^ neuronal somas and their axon terminals in the BLA. Optical fibers were implanted ipsilaterally in either the mPFC or BLA. After a 3-week recovery, yellow light (589 nm, 4-5 mW, constant stimulation; Thinker Tech Nanjing Co., Ltd., Nanjing, China) was delivered during stage 1 of the Y-maze test. Stage 2 was performed without light stimulation 1 h after the light stimulation.

AAV2/9-hSyn-SV40 NLS-Cre was injected unilaterally into the mPFC, and AAV2/2_Retro_-hEF1a-DIO-hChR2(H134R) was also injected ipsilaterally into the BLA. A guided cannula, which could be used for both optogenetic stimulation and micro-infusion, was implanted in the mPFC. After a 3-week recovery, QH (2 mM, 0.2 μL) was infused into the mPFC at a rate of 0.2 µL/min, and then the inner cannula was replaced by a customized optical fiber for optogenetics (Kedou Brain-Computer Technology Co. Ltd., Suzhou, China). The blue light, following the protocols described above, was delivered during stage 1 of the Y-maze test, 10 min after micro-infusion. Stage 2 was carried out without light stimulation 1 hour after stage 1. Mice were then euthanized for c-Fos staining 1 h after a 10-minute duration of light stimulation.

### Gene analysis in the mPFC region by RNA sequencing

Mice were deeply anesthetized with 2% isoflurane, and the mPFC regions were quickly dissected and frozen in liquid nitrogen. RNA sequencing service was provided by MajorBio (Shanghai, China). Total RNA was extracted from frozen tissues using Trizol reagent (Invitrogen, Carlsbad, CA, USA), followed by a standardized procedure including mRNA purification, mRNA fragmentation, cDNA synthesis, library construction, and paired-end sequencing (PE150) on an Illumina Novaseq^TM^ 6000. The raw data in FASTQ format were subsequently used for genome alignment (GRCm38) using Hisat2, sorting with SAMtools, and quantifying via FeatureCount. The generated count matrix was then used for the analysis of differentially expressed genes (DEGs) using the DESeq2 R package 24, 25. DEGs were defined as having a fold change > or < 1.5 between groups and an adjusted p-value < 0.05. Gene set annotations were conducted in Enrichr, followed by visualization using the ggplot2 R package. The gene-gene interaction network was constructed in STRING using the upregulated DEGs, followed by the identification of condensed sub-networks using MCODE 26 and visualization using Cytoscape.

### Immunohistochemistry

Mice were anesthetized with 2% isoflurane and then perfused with PBS, followed by 4% paraformaldehyde (PFA, Merck, Darmstadt, Germany). Brains were removed and post-fixed in 4% PFA for 12 h, followed by dehydration in a 30% sucrose solution for 24 h. Frozen slices were sectioned to a thickness of 30 µm or 20 µm (for STED) using a Thermo Fisher cryostat microtome (NX50, Waltham, Massachusetts, U.S.) and stored in an anti-icing solution at -20 °C. Slices were washed three times with PBS, each wash lasting 10 min, then blocked with immunofluorescence blocking solution (P0260, Beyotime Biotechnology Co., Shanghai, China) for 1 h. The blocking solution was washed off with three 10-minute PBS washes and incubated overnight at 4 °C with primary antibodies: mouse anti-Dopamine D2 receptor (1:200; sc-5303, Santa Cruz), rabbit anti-CaMKIIα (1:500; ab5683, Abcam), or guinea pig anti-c-Fos (1:500; 226308, synaptic system). Afterward, slices were washed three times with PBS and incubated with secondary antibodies (1:1000, goat anti-mouse 488, A21141 or goat anti-rabbit 568, A11011 or goat anti-guinea pig 568, A11075 or goat anti-guinea pig 488, A11073, Invitrogen; 1:200, goat anti-mouse Abberior STAR RED, STRED-100UG, Abberior; 1:200, goat anti-rabbit 555, 715-565-150, Jackson) for 2 h at 37 °C. Subsequently, all slices were mounted on slides with an anti-fade mounting medium containing DAPI (P0131, Beyotime Biotechnology Co., Shanghai, China) and kept at -20 °C. Confocal images were acquired under identical settings using a TCS SP8 confocal microscope (Leica, Wetzlar, Germany), and further image processing was done using LAS X software (Leica). STED images were acquired using the Abberior Facility Line (Abberior Instruments GmbH, Göttingen, Germany) fluorescence microscope integrated with a motorized inverted microscope IX83 (Olympus UPlanXAPO 60x, NA1.42, Tokyo, Japan). Abberior Star Red and Alexa 555 were used for imaging. A 775 nm STED laser was applied for Abberior Star Red, while a 595 nm STED laser was used for Alexa 555. Image processing was done with Lightbox 2024.8.19709-gf5c3940717 software (Abberior Instruments GmbH, Germany) and ImageJ 1.53k software (Wayne Rasband, National Institutes of Health, USA).

### Statistical analysis

The sample size estimation was based on our pilot study of a significant decrease in the novel arm preference of CSD mice from the baseline 41.79 (14.56) to 21.03 (6.55). To achieve a desired power of 80% and with a type I error set at 0.05, a minimum sample size of 6 mice per group is needed. Therefore, 6-9 mice per group were used for subsequent behavioral experiments. Data were represented as mean ± standard error of the mean (SEM). Normality was assessed using the Shapiro-Wilk test. For comparisons between two groups, the F-test was first used to check the equality of variances. If variances were equal, Student's* t*-test was applied; otherwise, Welch's *t*-test was used. For comparisons among multiple groups, one-way ANOVA followed by Tukey's post hoc test was performed. For repeated measures data, two-way repeated measures (RM) ANOVA was conducted, followed by Fisher's LSD or Šidák's multiple comparisons test. Effect sizes were calculated using G*Power 3.1 software (Germany). All statistical analyses were performed using GraphPad Prism 9.5.0 (GraphPad Software, San Diego, CA, USA). Statistical significance was defined as p < 0.05.

## Results

### Chronic sleep deprivation induced spatial memory impairment

**Figure 1:**
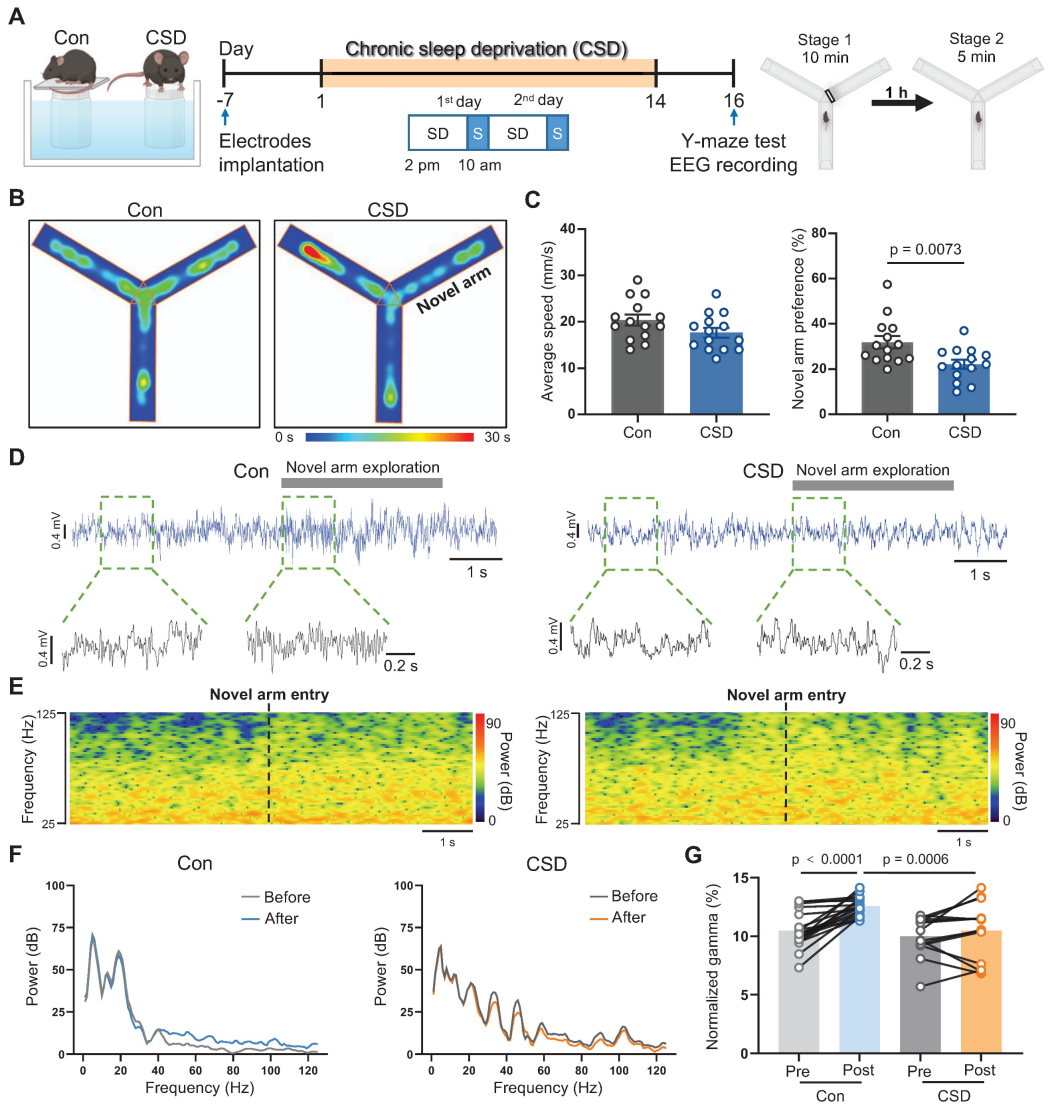
** Chronic sleep deprivation induced learning and memory impairment. (A)** Left, schematic drawings of the REM sleep deprivation. Middle, experimental design. Right, Schematic drawings of the Y-maze test. Con, the control group; CSD, the chronic sleep deprivation group; SD, sleep deprivation; S, sleep; EEG, electroencephalogram.** (B)** Representative heatmap tracing during the Y-maze test. **(C)** Quantification of average speed and novel arm preference index. **(D)** Representative signals of EEG before and after the mice entered the novel arm of the Control and CSD groups.** (E)** Representative spectrogram of the gamma oscillations before and after the mice entered the novel arm between the Control and CSD groups. **(F)** Representative traces of power spectral density of the EEG before and after the mice entered the novel arm between the Control (left) and CSD (right) groups.** (G)** Quantification of change in average gamma oscillation pre- and post-novel arm entry. For the Y-maze test, n = 14 mice for each group; For EEG recording, n = 19 trials from 7 mice for the Control group, n = 14 trials from 9 mice for the CSD group.

To explore the effects of sleep disorders on learning and memory, C57BL/6J mice were subjected to chronic sleep deprivation (CSD) for 2 weeks as reported previously 27 (Figure 1A). Mice were monitored hourly during a sleep deprivation period from 2 pm to 2 pm of the next day (Figure S1 and S2). This model regimen induced an increase in wakefulness along with a decrease in REM sleep while NREM sleep remained unchanged (Figure S1C-H) during the sleep deprivation period from 2 pm to 10 am of the next day. During the subsequent 4-hour recovery period from 10 am to 2 pm (Figure S2A-B), CSD mice exhibited a rebound in REM sleep, without significant changes in wakefulness or NREM sleep (Figure S2C-H). Moreover, analysis of the total 24-hour duration of sleep/wake states demonstrated that, despite the REM sleep rebound during the recovery, CSD mice showed an overall increase in wakefulness (Figure S2I), comparable levels of NREM sleep (Figure S2J), and a decrease in REM sleep (Figure S2K), indicating that the total REM sleep was reduced in this model.

We next sought to assess the impact of sleep deprivation on learning and memory using the Y-maze test (Figure 1A). Locomotor function was not affected following CSD. However, following a 2-day recovery from CSD, the preference for the novel arm in the CSD mice was decreased when compared to that of the controls (22% vs 32% of the Con group, t (26) = 2.91, p = 0.0073, Cohen's d = 1.10) (Figure 1B-C), indicating a decline in spatial memory after CSD. Previous studies demonstrated that gamma oscillations are critical for learning and memory 28. We further investigated whether gamma oscillations are involved in spatial memory encoding following CSD (Figure 1D-F). During the Y maze test, control mice entering the novel arm showed an increase in gamma oscillation power (t (31) = 5.93, p<0.0001; Figure 1G). However, mice subjected to CSD had a decrease in gamma oscillation power compared to the controls after entering the novel arm (t (62) = 3.60, p = 0.0006; Figure 1G). These findings suggest that CSD may chronically reduce REM sleep, leading to spatial memory impairment in mice.

### Chronic sleep deprivation increased Drd2 expression in the mPFC

**Figure 2:**
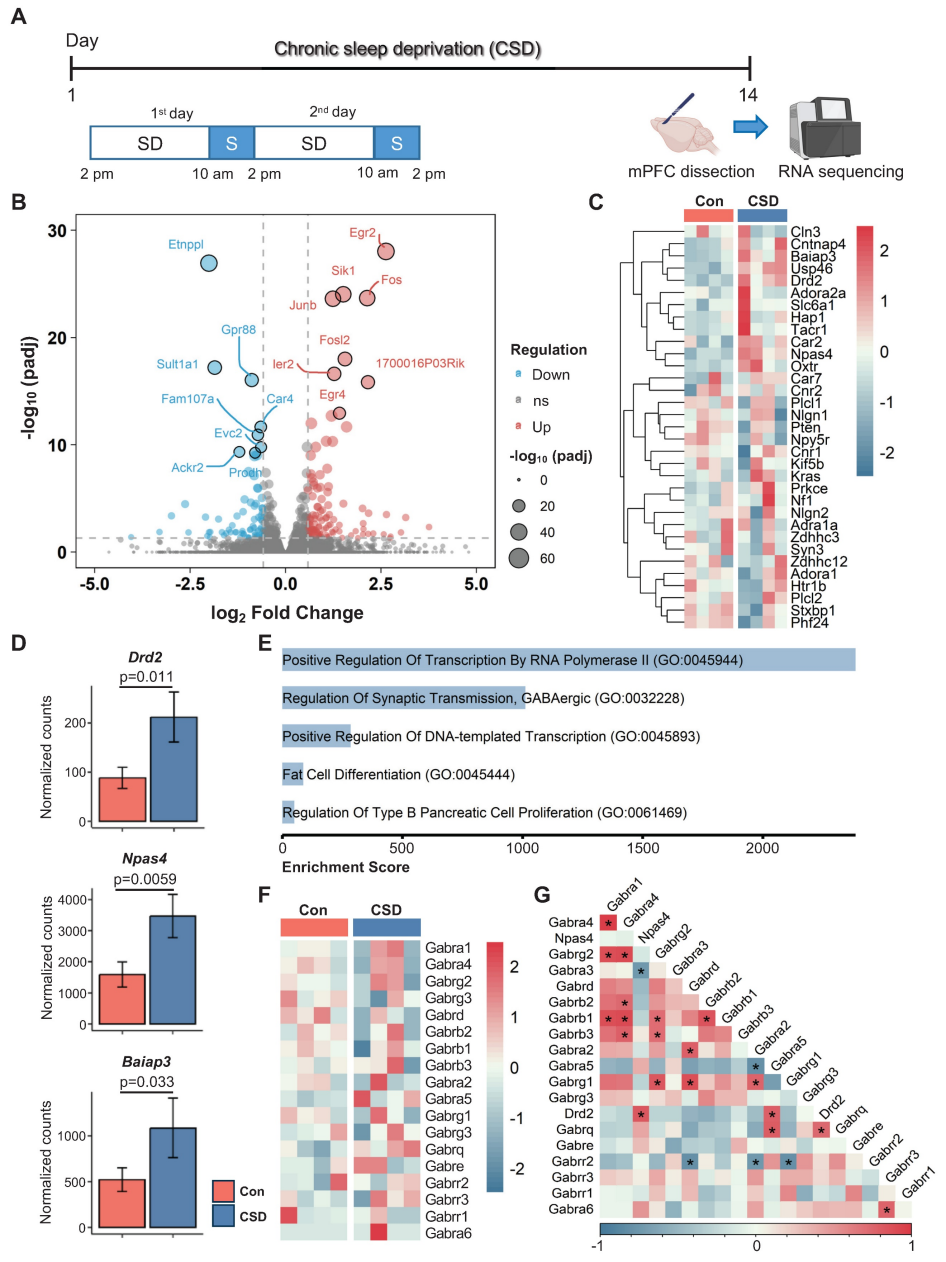
** Chronic sleep deprivation increased dopamine D2 receptor gene expression in the mPFC. (A)** Experimental design. SD, sleep deprivation; S, sleep.** (B)** Volcano plot of mPFC gene expression after CSD. padj, adjusted p-value; ns, no significance. **(C)** Heatmaps showing differentially expressed genes in the Con and CSD groups. Con, the control group; CSD, the chronic sleep deprivation group.** (D)** Quantification of *Drd2*, *Npase4*, and *Baiap3* gene expression between the two groups. **(E)** Gene ontology functional analysis diagram for the CSD group. **(F)** Heatmaps showing the gene expression of GABA receptor subunits (*Gabrs*) in the Con and CSD groups. **(G)** Heatmaps showing the correlation of Drd2, *Npase4, and Gabrs.* *p < 0.05. n = 4 mice for each group.

The mPFC plays a critical role in cognitive function. Insufficient sleep disrupts mPFC functional connectivity with cortical and subcortical regions, contributing to cognitive deficits 10. To elucidate the cellular mechanisms behind CSD-induced cognitive impairment, we conducted bulk RNA sequencing (RNA-seq) on the dissected mPFC from CSD mice in comparison with the controls (Figure 2A). We identified a total of 197 differentially expressed genes (DEGs, defined as a fold change > 1.5 between groups with an adjusted p < 0.05) in the CSD group, including 118 upregulated and 79 downregulated genes compared to the control group (Figure 2B). CSD led to an increase in gene expression levels of the dopamine D2 receptor (*Drd2*), neuronal PAS domain protein 4 (*Npas*4), and brain-specific angiogenesis inhibitor I-associated protein 3 (*Baiap3*) (Figure 2C-D). In contrast, the expression levels of other dopamine receptors, including *Drd1*, *Drd4,* and *Drd5*, remained unchanged following CSD (Figure S3A-C). Further gene ontology (GO) analysis revealed that the DEGs were enriched in the process of regulation of synaptic GABAergic transmission (Figure 2E), indicating an increase in inhibitory neurotransmission in the mPFC after CSD. Although CSD did not alter the expression of the GABA receptor subunits (*Gabrs*) genes directly (Figure 2F), Drd2, as a key modulator of GABAergic signaling, showed positive correlations with both the *Gabra5* and *Npas4* (Figure 2G). To further investigate functional gene relationships, we constructed gene-gene interaction networks with the upregulated DEGs. Molecular complex detection (MCODE) was then applied to unveil three highly interconnected subnetworks. Interestingly, the largest subnetwork included Drd2 as a key node with high connectivity. The genes within this subnetwork were enriched for Drug targets in autistic disorder and Drd2 expression targets (Figure S3D-E). In the other two subnetworks, genes involved in the cell cycle and astrocyte functions were identified, suggesting the potential involvement of astrocyte reactivation (Figure S3D-E).

Drd2 was reported to be predominantly expressed in pyramidal neurons in the mPFC 29. To further delve into the distribution of Drd2 in the mPFC, we co-labeled Drd2 with the pyramidal neuron marker CaMKIIα and found that approximately 85% of Drd2 was co-localized with CaMKIIα (Figure S4A-C). These were both in layers II/III and V/VI of the mPFC (Figure S4E). Similar to *Drd2* gene expression in the bulk RNA-seq following CSD, the increased Drd2 was found primarily in layer II/III (Drd2 fluorescence intensity 3462 vs 6970 of the Con group, p = 0.0026) (Figure S4D).

Next, we investigated whether CSD affects GABAergic transmission by *ex vivo* brain slice electrophysiology recording of miniature inhibitory postsynaptic currents (mIPSCs) in CaMKIIα⁺ neurons of the mPFC. To specifically target these pyramidal neurons, AAV-CaMKIIα-mCherry was injected into the mPFC. Following viral transduction, mice were subjected to the CSD protocol (Figure S5A-B). *Ex vivo* electrophysiological recordings showed that CSD significantly increased the frequency of mIPSCs but not the amplitude (Figure S5C-F). Together, these results suggest that CSD may lead to upregulation of Drd2 expression, thereby enhancing GABAergic synaptic transmission onto CaMKIIα⁺ neurons in the mPFC.

### Dopamine input to the mPFC decreased during REM sleep

Given that Drd2 is the primary target of the dopamine (DA) system, we examined extracellular DA levels across the sleep-wakefulness cycle in the mPFC using a G protein-coupled receptor (GPCR) activation-based sensor for DA (GRAB_DA_). When transduced GRAB_DA_ expressed in mPFC neurons was detected using implanted optical fibers for photometry. With this system, we were able to examine the relationship between DA release and sleep-wakefulness state transition (Figure S6A). We found that DA release decreased from NREM to REM sleep, and increased from REM sleep to NREM sleep (Figure S6B-E). Intriguingly, DA release increased during the REM-to-wakefulness transitions and remained stable from the wakefulness-to-NREM sleep transitions (Figure S6B-E), suggesting that REM sleep may suppress DA input to the mPFC. Next, we explored whether CaMKIIα^+^ neuronal activity was modulated in accordance with DA fluctuations during REM sleep by transducing the mPFC with AAV9-CaMKIIα-GCaMP7f and subsequently conducted fiber photometry (Figure S7A). Calcium influx was decreased during transitions from NREM to REM sleep and from wakefulness to NREM sleep, while it was increased during transitions from REM sleep to wakefulness and from REM to NREM sleep (Figure S7B-E). Taken together, the reduction in DA input to the mPFC was accompanied by decreased CaMKIIα⁺ neuronal activity during REM sleep, suggesting that DA may play a key role in regulating CaMKIIα⁺ neuronal activity.

### Drd2 was involved in learning and memory impairment induced by chronic sleep deprivation

**Figure 3:**
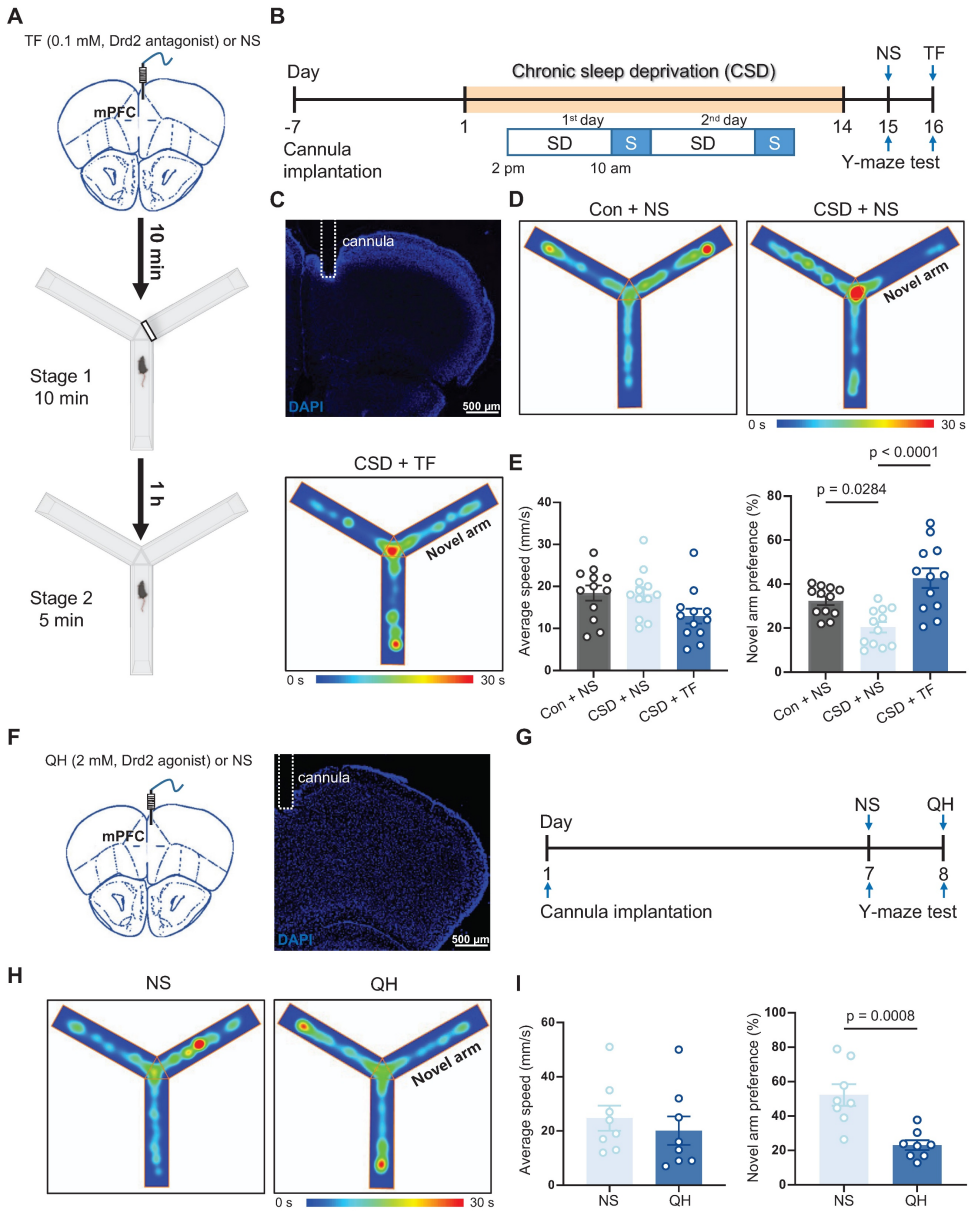
** Drd2 was involved in learning and memory impairment induced by chronic sleep deprivation. (A)** Schematic of the micro-infusion and the Y-maze test. The black line rectangle represents a barrier to close the novel arm during stage 1 of the Y-maze test. mPFC, medial prefrontal cortex; TF, Trifluoperazine 2HCl; NS, normal saline. **(B)** Experimental design. SD, sleep deprivation; S, sleep. **(C)** Representative image showing the position of a cannula in the mPFC. **(D)** Representative tracing heatmaps during the Y-maze test. **(E)** Quantification of average speed (left) and novel arm preference index (right). Con, the control group; CSD, the chronic sleep deprivation group. n = 12 mice for each group. **(F)** Schematic of the micro-infusion (left) and representative image showing the position of a cannula in the mPFC (right). **(G)** Experimental design. **(H)** Representative tracing heatmaps during the Y-maze test. **(I)** Quantification of average speed (left) and novel arm preference index (right). QH, quinpirole hydrochloride. n = 8 mice for each group.

Next, we investigated whether Drd2 is essential for CSD-induced learning and memory impairment through micro-infusion of a Drd2 antagonist, Trifluoperazine 2HCl (TF), into the mPFC 10 min before the Y-maze test (Figure 3A-C). TF did not affect movement but successfully reversed the impaired preference for moving into the novel arm in the CSD male mice compared to normal saline treatment (43% vs 20% of the CSD + NS group, q (33) = 7.12, p < 0.0001, Cohen's d = 1.79; 20% vs 32% of the Con + NS group, q (33) = 3.82, p = 0.0284, Cohen's d = 1.57) (Figure 3D-E). To further elucidate the role of Drd2 in cognitive function, we administered a Drd2 agonist, quinpirole hydrochloride (QH), into the mPFC of naïve mice to evaluate their performance in the Y-maze test (Figure 3F-G). QH significantly decreased the preference index for the novel arm (23% vs 52% of the NS group, t (14) = 4.25, p = 0.0008, Cohen's d = 2.12) (Figure 3H-I), suggesting the cognitive impairment induced by CSD.

In addition, we investigated whether Drd2 in the mPFC also plays a role in learning and memory in female mice. Similar to male mice, CSD significantly reduced the preference index for the novel arm in female mice (Figure S8A-E), indicating impaired spatial memory. Notably, micro-infusion of TF into the mPFC reversed this deficit (Figure S8A-E), increasing the preference index compared to CSD mice treated with normal saline.

We further examined whether Drd2 participates in the sleep-wakefulness cycle. QH or TF was specifically administered to the mPFC in the naïve mice, and the EEG/EMG was recorded continuously for 24 h from zeitgeber time (ZT) 0 to ZT 24 (Figure S9A-D). Both QH and TF reduced wakefulness and increased NREM sleep during the first hour of recording, suggesting that high-concentration Drd2 antagonists or agonists may promote NREM sleep by disrupting local wake-promoting circuitry post-infusion (Figure S9E-G). However, no significant changes were observed in another hourly distribution of wakefulness, NREM, and REM sleep across the remaining recording period (Figure S9E-G), nor in the total wakefulness, NREM sleep, and REM sleep duration (Figure S9H-J). Our findings suggest that while Drd2 in the mPFC may be crucial for learning and memory processes, it does not seem to be involved in regulating the sleep-wake cycle.

### CaMKIIα^+^ neuronal activation in the mPFC rescued learning and memory impairment induced by chronic sleep deprivation

**Figure 4:**
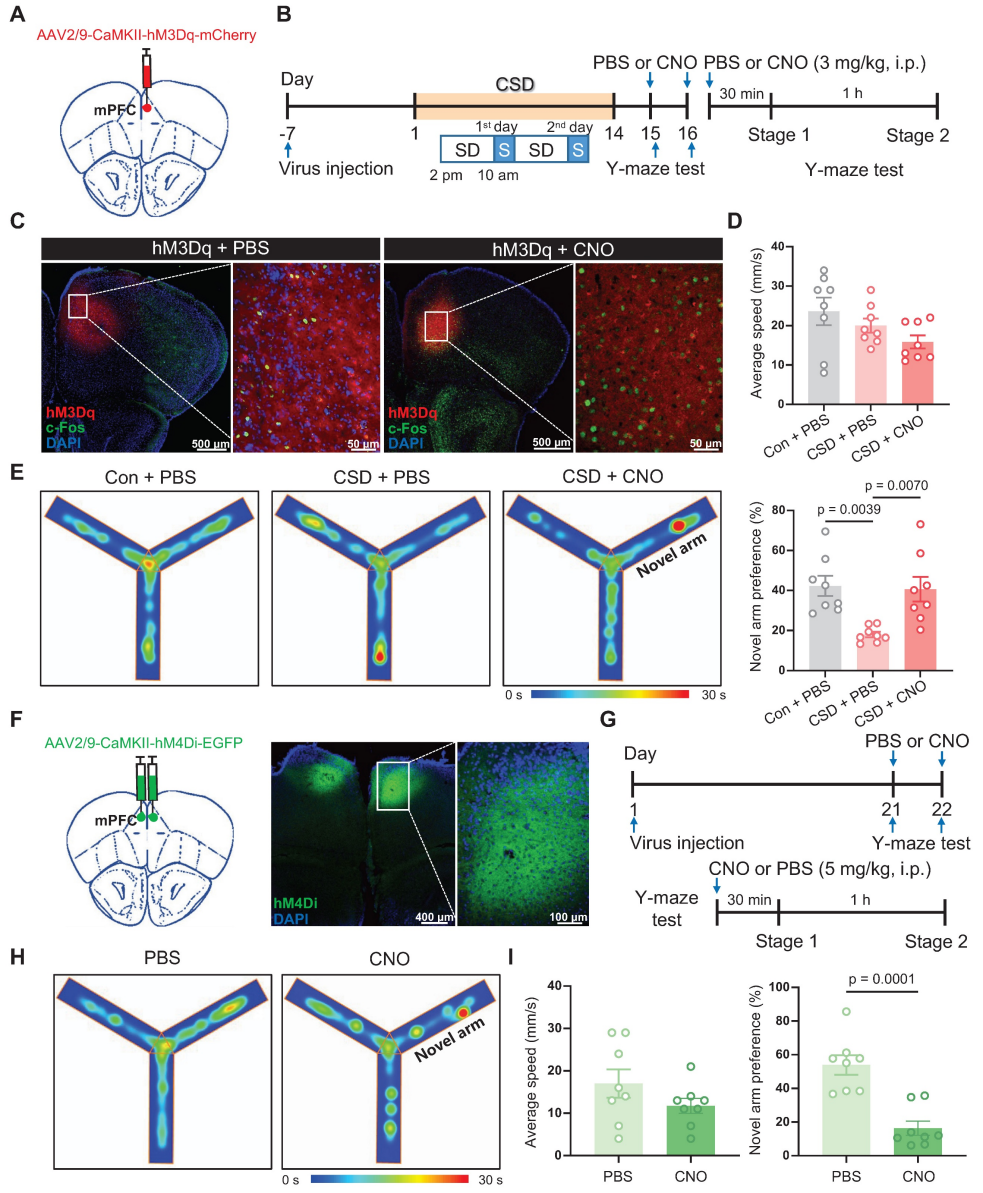
** Activation of CaMKIIα^+^ neurons rescued learning and memory impairment induced by chronic sleep deprivation. (A)** Schematic of virus injection. mPFC, medial prefrontal cortex.** (B)** Experimental design. SD, sleep deprivation; S, sleep; PBS, phosphate buffer saline; CNO, Clozapine N-oxide.** (C)** Representative coronal brain section stained with c-Fos antibody (green), hM3Dq (red), and DAPI (blue), and high-magnification image of the white-boxed area. Left, the basal c-Fos level; Right, the c-Fos level after hM3Dq activation. **(D)** Quantification of average speed (top) and the novel arm preference index (bottom). Con, the control group. n = 8 mice for each group. One mouse was excluded due to a missed viral.** (E)** Representative tracing heatmaps during the Y-maze tests. **(F)** Left, Schematic drawing of virus injection. Middle, Representative coronal brain section stained with hM3Dq (green) and DAPI (blue). Right, Representative high-magnification image of the white-boxed area of the middle image. **(G)** Experimental design. **(H)** Representative tracing heatmaps during the Y-maze tests. **(I)** Quantification of average speed (left) and the novel arm preference index (right). n = 8 mice for each group.

Given that Drd2 is highly co-localized with CaMKIIα in the mPFC, and CSD disrupts neuronal activity and synaptic transmission of CaMKIIα^+^ neurons 9, 29. We further asked whether CaMKIIα^+^ neurons play a crucial role in reversing learning and memory impairment induced by CSD. To monitor the activity of CaMKIIα^+^ neurons during the Y-maze test, GCaMP7f was introduced to CaMKIIα^+^ neurons in the mPFC under the CaMKIIα promoter and assessed with fiber photometry imaging (Figure S10A). The mPFC CaMKIIα^+^ neuronal activity increased when mice began to cross into the novel arm (0.87 vs -0.00017, p = 0.0002) (Figure S10B-D). Also, to further confirm that activation of CaMKIIα^+^ neurons is causally involved in learning and memory, the modified human muscarinic acetylcholine receptor type 3 (hM3Dq) that will increase neuronal activity was specifically expressed in CaMKIIα^+^ neurons of the mPFC (Figure 4A-B).

The receptor agonist Clozapine-N-oxide (CNO) was administered i.p., preferentially increasing neuronal activity with the increase of c-Fos expression (Figure 4C). Following CSD, mice treated with CNO before the Y-maze test showed a greater preference for moving into the novel arm than mice treated with PBS (41% vs 18% of the PBS group, q (21) = 4.834, p = 0.007, Cohen's d = 1.79) (Figure 4D-E). We further inhibited the activity of CaMKIIα^+^ neurons in the mPFC through bilateral injection of human muscarinic receptor 4 (hM4Di) (Figure 4F). After a 21-day recovery, the mice without sleep deprivation were subjected to perform the Y-maze test upon intraperitoneal injection of PBS or CNO (Figure 4G). Inhibition of CaMKIIα^+^ neurons with CNO significantly reduced the preference for the novel arm compared to mice treated with PBS (16% vs 54% of the PBS group, t (14) = 5.25, p = 0.0001, Cohen's d = 2.63) (Figure 4H-I). These findings suggest that CaMKIIα⁺ neurons in the mPFC may be critical for spatial memory, particularly in the context of CSD-induced memory impairment.

Next, we investigated whether the CaMKIIα^+^ neurons in the mPFC are crucial for sleep regulation. We injected AAV-CaMKII-mCherry or AAV-CaMKII-hM4Di specifically into the mPFC and conducted sleep recordings in the mCherry- or hM4Di-expressing mice without sleep deprivation on day 21-22 from 2 pm to 6 pm (Figure S11A). Hourly recordings revealed no significant differences in the proportions of wakefulness and NREM sleep between hM4Di-expressing mice treated with PBS vs CNO (Figure S11B-E). However, REM sleep was significantly reduced following CNO administration (Figure S11B-E). Furthermore, the total duration of wakefulness and NREM sleep states did not differ significantly between the CNO-treated and PBS-treated mice, but REM sleep was reduced in the CNO-treated group of hM4Di-expressing mice (2.2% vs 7.1% of the hM4Di + PBS group, t (12) = 4.24, p = 0.0011, Cohen's d = 2.48; 2.2% vs 7.2% of the mCherry + CNO group, t (24) = 4.73, p < 0.0001, Cohen's d = 2.89) (Figure S11F-H). In contrast, no significant changes were observed in either hourly or total sleep-wake states in mCherry-expressing mice between PBS and CNO treatments (Figure S11B-H). Our findings suggest that CaMKIIα⁺ neurons may play a specific role in regulating REM sleep, but not NREM sleep or wakefulness.

### Drd2 regulation of the mPFC-BLA circuit contributed to learning and memory impairment induced by chronic sleep deprivation

To investigate the target brain regions innervated by the CaMKIIα^+^ neurons of the mPFC, we examined their axonal projections *via* anterograde tracing (Figure S12A). Axons of EGFP-expressing CaMKIIα^+^ neurons were broadly distributed to various subcortical regions, including the forceps minor of the corpus callosum (fmi), dorsal striatum (dSTR), genu of the corpus callosum (gcc), accumbens nucleus core (AcbC), lateral globus pallidus (LGP), paratenial thalamic nucleus (PT), reticular thalamic nucleus (Rt), internal capsule (ic), mediodorsal and ventromedial thalamic nucleus, medial globus pallidus (MGP), basolateral amygdala (BLA), lateral hypothalamus (LH), parasubthalamic nucleus (PSTh) and substantia nigra, reticular part (SNR) (Figure S12B-L).

**Figure 5:**
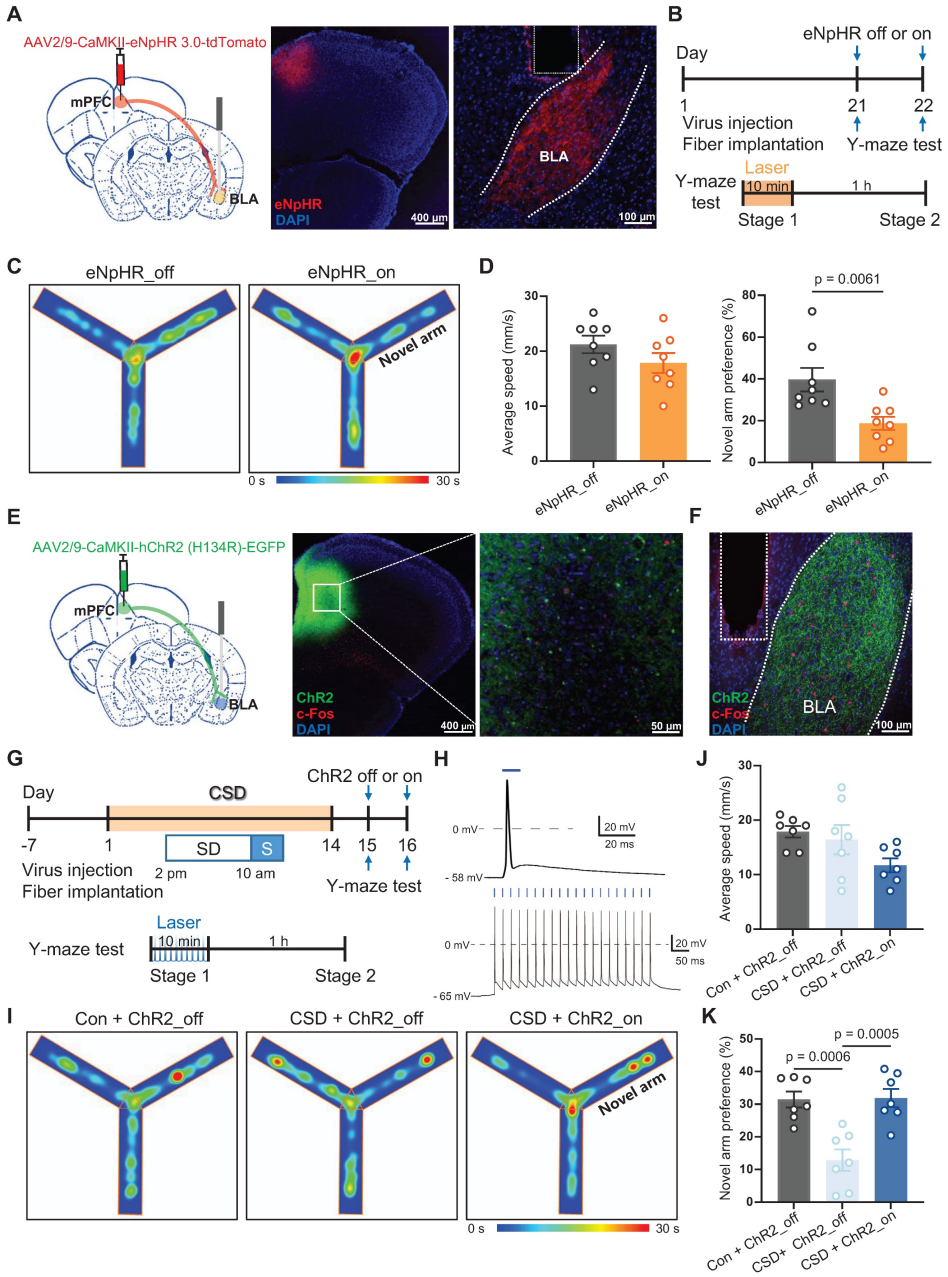
** The mPFC-BLA circuit contributed to learning and memory impairment induced by chronic sleep deprivation. (A)** Schematic of virus injection and optical fiber implantation (left). Representative coronal brain sections of the mPFC (middle) and BLA (right), stained with eNpHR (red) and DAPI (blue). mPFC, medial prefrontal cortex; BLA, basolateral amygdala.** (B)** Experimental design. **(C)** Representative tracing heatmaps during the Y-maze tests. **(D)** Quantification of average speed (left) and the novel arm preference index (right). eNpHR_on or eNpHR_off, the group with or without eNpHR stimulation. n = 8 mice for the group with or without eNpHR stimulation.** (E)** Left, Schematic drawing of virus injection and optical fiber implantation. Representative coronal brain sections of the mPFC stained with c-Fos antibody (red), ChR2 (green), and DAPI (blue) (right). **(F)** Representative coronal brain sections of the BLA stained with c-Fos antibody (red), ChR2 (green), and DAPI (blue). **(G)** Experimental design.** (H)** A single 10-ms light pulse (top) and a train of 1-ms pulses at 20 Hz (bottom) for the light-evoked action potentials of mPFC CaMKII^+^ neurons. **(I)** Representative tracing heatmaps during the Y-maze tests. Con, the control group; ChR2_on or ChR2_off, the group with or without ChR2 stimulation.** (J, K)** Quantification of average speed and the novel arm preference index. n = 7 mice for each group. One mouse was excluded due to an outlier.

Notably, BLA is an important nucleus of the memory network, including memories with emotionally arousing experiences and object recognition memory 30, 31. We explored whether the BLA is a functional downstream target of the mPFC in mediating spatial memory processing. NpHR is a light-sensitive chloride channel that silences neuronal activity by inducing hyperpolarization. We used an AAV vector expressing eNpHR3.0-tdTomato under the control of the CaMKII promoter to selectively inhibit CaMKII-positive neurons in the mPFC. We injected AAV-CaMKII-eNpHR-tdTomato into the mPFC and implanted an optic fiber into the BLA (Figure 5A-B). The inhibition of mPFC projections to BLA significantly reduced the preference for the novel arm during the Y-maze test (19% vs 40% of the eNpHR_off group, t (14) = 3.23, p = 0.0061, Cohen's d = 1.61) (Figure 5C-D). In contrast, AAV-CaMKII-ChR2-EGFP was specifically injected into the mPFC, and an optical fiber was implanted in the BLA (Figure 5E-G). Blue light stimulation effectively induced action potentials in CaMKII^+^ neurons in mPFC brain slices (Figure 5H). Furthermore, activation of mPFC projections to BLA reversed the reduced preference for the novel arm caused by CSD (32% vs 13% of the ChR2_off group, q (18) = 6.68, p = 0.0005, Cohen's d = 2.37) (Figure 5I-K).

**Figure 6:**
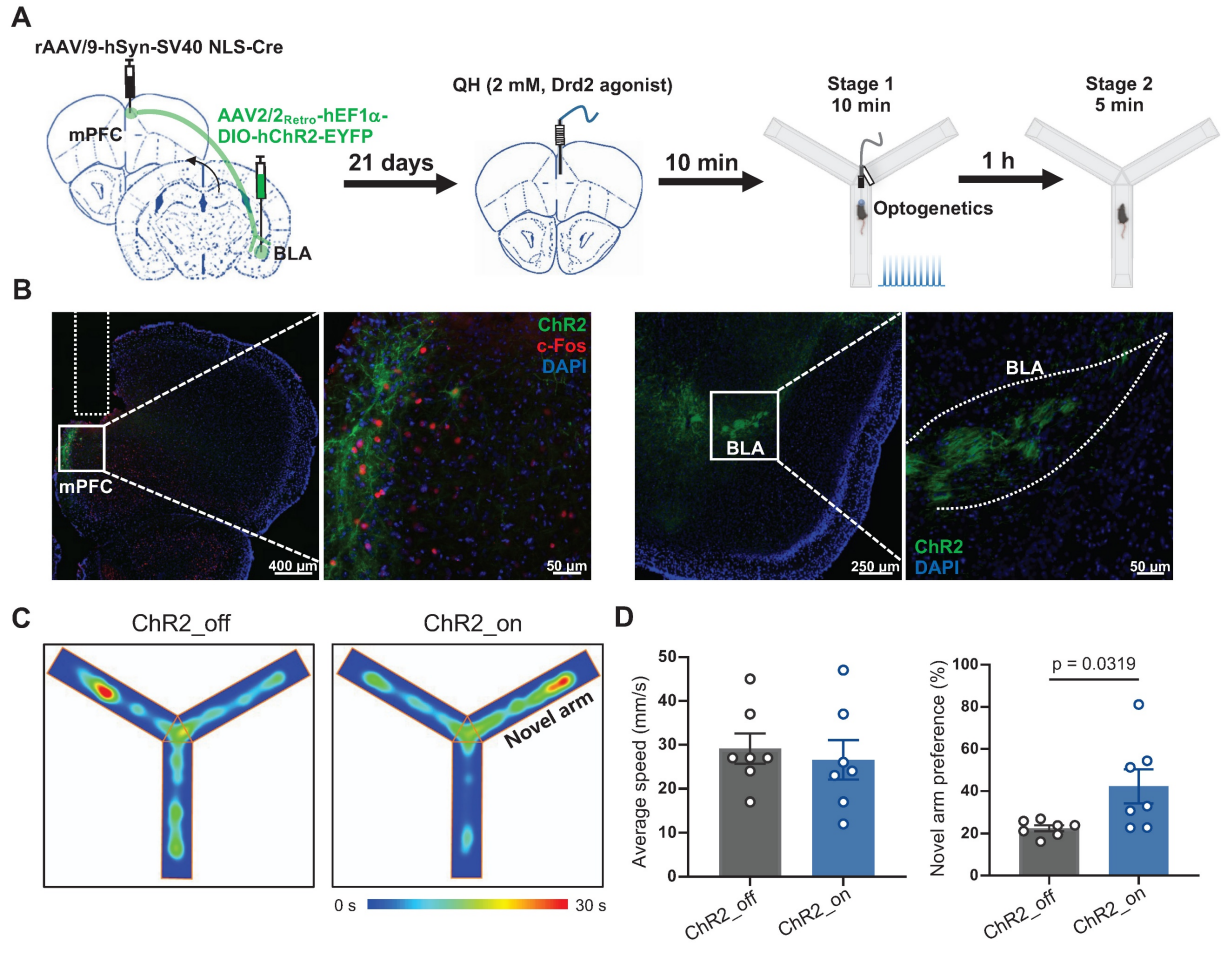
** Drd2-mediated mPFC-BLA circuit contributed to learning and memory impairment induced by chronic sleep deprivation. (A)** Experimental design and schematic of virus injection, optical fiber implantation, micro-infusion, and Y-maze test. **(B)** Representative coronal brain sections of the mPFC (left) and BLA (right) stained with c-Fos antibody (red), ChR2 (green), and DAPI (blue). **(C)** Representative tracing heatmaps during the Y-maze tests. **(D)** Quantification of average speed and the novel arm preference index. n = 7 mice for each group. mPFC, medial prefrontal cortex; BLA, basolateral amygdala. One mouse was excluded due to low viral expression.

To specifically determine whether Drd2 contributes to learning and memory impairment by regulating the mPFC-BLA circuit, AAV-hSyn-Cre was injected into the mPFC to drive Cre recombinase expression in local neurons, while retroAAV-hEF1-DIO-hChR2 was injected into the BLA. Retrograde transport of the virus enabled Cre-dependent expression of ChR2 in the specific mPFC neurons projecting to the BLA. A cannula was implanted in the mPFC to locally deliver the Drd2 agonist QH, which can be replaced by an optical fiber to allow optogenetic activation of the mPFC-BLA pathway (Figure 6A-B). On day 21, Drd2 agonist QH was administered to the mPFC in mice without sleep deprivation. We found that activation of the BLA-projecting neurons in the mPFC reversed the reduced preference for the novel arm caused by the Drd2 agonist (42% vs 22% of the ChR2_off group, t (12) = 2.43, p = 0.0319, Cohen's d = 1.30) (Figure 6C-D). These findings indicated that Drd2 specifically targeting BLA likely contributes to learning and memory impairment induced by chronic sleep deprivation.

## Discussion

In the present study, we demonstrated that normal REM sleep induced a decrease in dopamine input to the mPFC. In contrast, chronic REM sleep deprivation increased dopamine release, which, in turn, led to increased dopamine D2 receptor (Drd2) expression in the CaMKIIα^+^ neurons of the layers II/III of the mPFC. These resulted in insufficient neuronal output from the mPFC projection to the BLA during the acquisition of spatial memory and ultimately impaired cognitive performance. Our data further demonstrated that Drd2-mediated-CaMKIIα^+^ neuronal inhibition did not participate in sleep regulation but contributed to CSD-induced cognitive impairment. Furthermore, CaMKIIα^+^ neuronal activation rescued CSD-induced learning and memory decline. Our work indicates that the potential targeting of the Drd2-associated mPFC-BLA neural circuit may serve as a therapeutic strategy in alleviating the cognitive deficits that commonly occur in sleep disorder patients.

Dopamine (DA), released from midbrain neurons in the ventral tegmental area and the substantia nigra pars compacta, is involved in the regulation of sleep-wake states 32. Recent studies found that during the sleep-wake cycle, DA input had distinct dynamic patterns across different brain regions 33. In mice, a transient increase of DA in the BLA terminated NREM sleep and initiated REM sleep, whilst DA depletion diminished REM sleep 34. In line with our study, a dramatic drop of DA input in the mPFC was found during REM sleep, but not during the transition from wakefulness to NREM sleep 33.

REM sleep deprivation led to mPFC functional impairment and subsequently caused cognitive function decline. This aligns with earlier studies demonstrating that sleep deprivation caused memory decline and triggered anxiety and social deficiency in rodents 9, 10 and humans 35. In agreement, our transcriptome analysis revealed considerable DEGs in the mPFC following sleep deprivation, highlighting the essential role of normal DA dynamics in maintaining the physiological functions of the mPFC. DA signaling has long been considered to be associated with different types of memory. In the mouse model of Alzheimer's disease, cortical dopamine release was reduced during the acquisition of new object memory; restoring dopamine levels during this stage improved the performance of object recognition 36. The role of DA signaling in memory acquisition appears to be conserved across species, as evidenced by observations in Drosophila during the formation of olfactory memory, which requires the activation of the dDA1 receptor, a homolog of the mammalian Drd1 37. In humans, single-nucleotide polymorphisms (SNPs) of Drd2 that are associated with high PFC activity during working memory tasks were reported to have better cognitive performance 38. However, Drd2 deletion in the mouse hippocampus reduced the acquisition and consolidation of spatial and associative forms of memory 39, 40. In our study, the annotation of DEGs implied an increased inhibitory synaptic neurotransmission, with Drd2 identified as one of the upregulated genes after REM sleep deprivation. This indicates that REM sleep disruption likely results in abnormal Drd2 signaling in the mPFC. Functionally, we showed that the Drd2 agonist QH significantly impaired spatial memory, whereas the Drd2 antagonist TF exhibited improvement in cognition after sleep deprivation. Therefore, the elevation of Drd2, which may lead to increased Drd2 signaling and decreased neuronal excitability in the mPFC, serves as an underlying molecular mechanism for abnormal spatial memory processing following sleep deprivation.

Memory formation and encoding require communication within the networks comprising various brain regions, including the cortex, hippocampus, thalamus, and amygdala. BLA is a highly interconnected brain region that is reciprocally connected with the cortex and hippocampus. The BLA is recognized as a hub for modulating memory processing across different stages and types of memory. It is well-known that BLA is responsible for processing fear memories or memories associated with emotionally arousing experiences 41, 42. Additionally, the BLA is involved in the consolidation of object recognition memory through modulating neuronal transmission in the entorhinal or insular cortex 43. The BLA receives extensive projections from other brain regions, e.g., mPFC and locus coeruleus, for executing memory encoding and consolidation. A recent study reported that a functional connection from the mPFC subregion-prelimbic cortex (PrL) to the BLA facilitated acquisition 44 and extinction of fear memory 45, suggesting a role for the mPFC-BLA circuit in memory processing. However, the factors shaping this connection affecting memory acquisition are unknown. In this study, we demonstrated that activation of the mPFC-BLA connection through chemogenetics or optogenetics facilitates the acquisition of spatial memory. On the other hand, Drd2 signaling appears to gate the mPFC-BLA connection in modulating spatial memory processing. Drd2 activation in the mPFC is sufficient to impair spatial recognition, but activation of mPFC-BLA projection neurons abolishes this detrimental effect, as shown in our study. Our work is in line with a previous study showing that low but not high doses of Drd2 agonist quinpirole, administered systemically, jeopardized PFC cognitive function in young-adult monkeys 46. In contrast, human volunteers, received Drd2/3 antagonist sulpiride orally had planning and spatial working memory impairment 47. The reasons for these discrepancies are unknown. Undoubtedly, different subjects (human vs monkeys vs mice) and different administration routes (systemically vs locally) together with complicated actions of dopamine and its receptors in the central nervous system are all likely responsible.

Our study has several limitations. Firstly, the D2 receptor agonist was administered in mice without CSD, even though Drd2 expression is altered under CSD conditions. This mismatch may limit the applicability of the pharmacological findings to the CSD model. While the D2 receptor antagonist was tested in CSD mice, future studies should examine the effects of both agonists and antagonists with the same pathological context to ensure more accurate interpretation and translational relevance. Secondly, this study was mainly focused on male mice, although the phenotypic changes of female mice following CSD are similar to male gender. Neural circuits and even molecular changes are subjected to further study.

In summary, our data indicate that REM sleep deprivation disrupted sleep-related dopamine fluctuation, leading to increased Drd2 expression in CaMKIIα^+^ pyramidal neurons of the mPFC and subsequently hindered spatial memory processing. Selective activation of the mPFC-BLA projection improved REM sleep deprivation-induced spatial memory impairment. Our findings provided a neural circuit and molecular mechanism by which REM sleep deprivation increased Drd2 expression to attenuate mPFC-BLA connectivity and ultimately impair memory acquisition and encoding. Our work may highlight the potential of targeting Drd2 signaling in the mPFC with its antagonist *per se* for attenuating cognitive deficits commonly experienced in patients with sleep disorders.

## Supplementary Material

Supplementary figures and table.

## Figures and Tables

**Figure 7:**
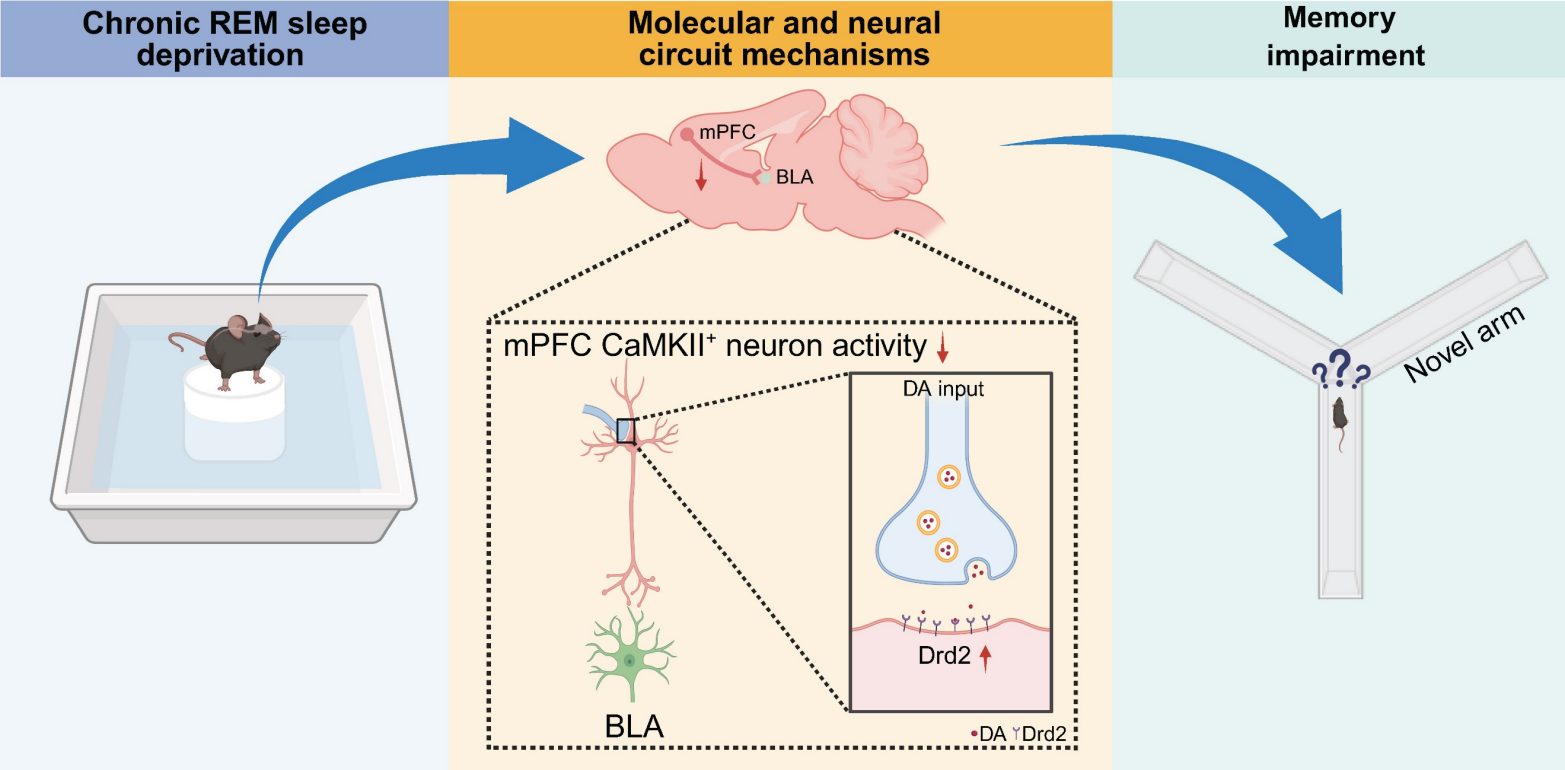
** Dopamine D2 receptor modulating mPFC-BLA circuit contributes to chronic sleep deprivation-induced memory impairment in mice.** Chronic REM sleep deprivation increased the expression of dopamine receptor 2 (Drd2) in CaMKIIα^+^ neurons of the medial prefrontal cortex (mPFC), leading to decreased neuronal activity, dysregulation of the mPFC-basolateral amygdala (BLA) circuit, and learning and memory impairment in mice.

## Abbreviations

CSD: chronic sleep deprivation
mPFC: medial prefrontal cortex
Drd2: dopamine D2 receptor
REM: rapid-eye movement
NREM: non-rapid-eye movement
CaMKII: Ca^2+^/calmodulin-dependent protein kinase II
BLA: basolateral amygdala
EEG: electroencephalogram
EMG: electromyogram
TF: Trifluoperazine 2HCl
QH: Quinpirole hydrochloride
mIPSC: miniature inhibitory postsynaptic current
SEM: standard error of the mean
CNO: Clozapine N-oxide
DEGs: differentially expressed genes
PFA: paraformaldehyde
ANOVA: analysis of variance
RNA-seq: RNA sequencing
GO: gene ontology
MCODE: molecular complex detection
DA: dopamine
GPCR: G protein-coupled receptor
GRAB_DA_: G protein-coupled receptor activation-based sensor for DA
hM3Dq: human muscarinic acetylcholine receptor type 3
hM4Di: human muscarinic receptor 4
PrL: prelimbic cortex
MO: medial orbital cortex
Cg1: cingulate cortex area 1
fmi: forceps minor of the corpus callosum
CPu: caudate putamen
gcc: genu of the corpus callosum
AcbC: accumbens nucleus, core
LGP: lateral globus pallidus
PT: paratenial thalamic nucleus
Rt: reticular thalamic nucleus
ic: internal capsule
MD: mediodorsal thalamic nucleus
VM: ventromedial thalamic nucleus
MGP: medial globus pallidus
LH: hypothalamus
PSTh: parasubthalamic nucleus
SNR: substantia nigra, reticular part
SNP: single nucleotide polymorphism
NS: normal saline
PBS: phosphate buffer saline
padj: adjusted p-value
ns: no significance
ZT: zeitgeber time
