# Supplementary material for: Dopamine D2 receptor modulating mPFC-BLA circuit contributes to chronic sleep deprivation-induced memory impairment in mice
Source: Theranostics. 2025 Aug 16;15(17):9073–90. doi: 10.7150/thno.114797 (PMC12439335; doi:10.7150/thno.114797)
Supplement: Supplementary file 1 — Supplementary figures and table. [file thnov15p9073s1.pdf]

**Yang J. et al. Dopamine D2 receptor modulating mPFC-BLA circuit contributes to chronic sleep deprivation-induced memory impairment in mice**

**Supplementary Table and Figures**

**Table S1. Details of viruses used in injections along with their function.**

| Name | Titer (vg/mL) | Company | Function |
|---|---|---|---|
| AAV2/9-hSyn-GRABeen DA2m-WPRE-pA | $1.15\times10^{13}$ | Taitool, China | Photometry |
| AAV2/9-CaMKIIα-GCaMP7f-WPRE-pA | $1.12\times10^{13}$ | Taitool, China | Photometry |
| AAV2/9-CaMKIIα-hM3Dq-mCherry-WPRE-pA | $1.44\times10^{13}$ | Taitool, China | Chemogenetics |
| rAAV2/9-CaMKIIα-EGFP-WPRE-pA | $3.75\times10^{12}$ | BrainCase, China | Neural tracing |
| AAV2/9-CaMKIIα-hChR2-EGFP-WPRE-pA | $1.48\times10^{13}$ | Taitool, China | Optogenetics |
| AAV2/5-CaMKIIα-eNpHR-tdTomato-WPRE-pA | $1.40\times10^{13}$ | Taitool, China | Optogenetics |
| rAAV/9-hSyn-SV40 NLS-Cre | $1.12\times10^{13}$ | BrainCase, China | Cre expression |
| AAV2/9-CaMKIIα-hM4Di-EGFP-ER2-WPRE-pA | $1.44\times10^{13}$ | Taitool, China | Chemogenetics |
| AAV2/$2_{Retro}$-hEF1α-DIO-hChR2-EYFP-WPRE-pA | $1.15\times10^{13}$ | Taitool, China | Optogenetics |
| AAV2/9-mCaMKIIα-mCherry-ER2-WPRE-pA | $5.05\times10^{12}$ | BrainCase, China | Labelling/Control |

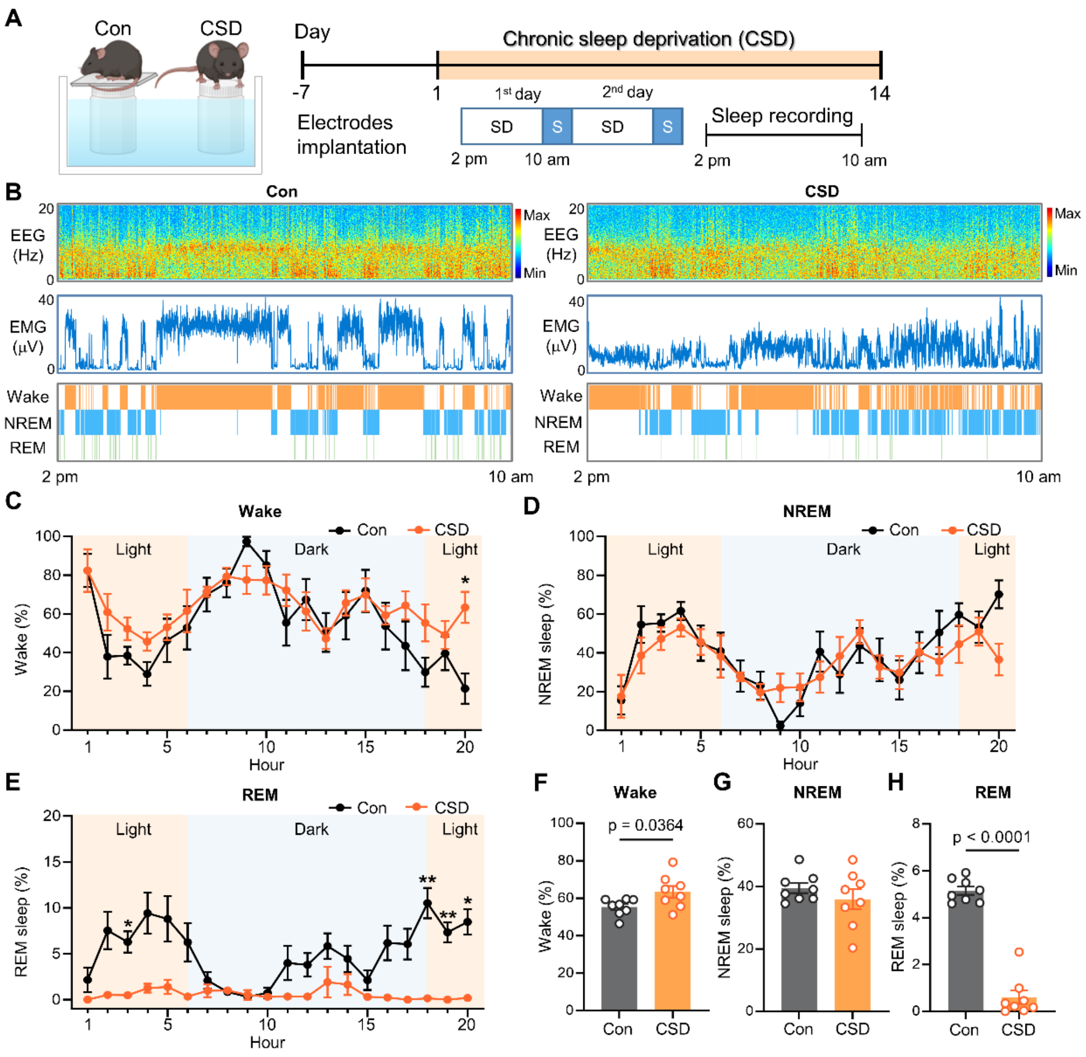


**Figure S1. The sleep-wake patterns during 20-h sleep deprivation period. (A)** Left, Schematic drawings of the sleep deprivation model. Right, Experimental design. Con, the control group; CSD, the chronic sleep deprivation group; SD, sleep deprivation; S, sleep. **(B)** Representative signals for EEG (upper), EMG (middle), and sleep architecture (bottom) during the 20-h period of sleep deprivation between the Control (left) and CSD (right) groups. EEG, electroencephalogram; EMG, electromyography; NREM, non-rapid eye movement; REM, rapid eye movement. **(C–E)** Representative graph of the hourly percentage of brain states from 2 pm to the next day at 10 am. **(F–H)** The total percentage of each brain state over a 20-h duration of sleep deprivation. Data are presented as mean ± SEM. *p < 0.05, **p < 0.01. n = 8 mice for each group; two mice were excluded prior to analysis due to poor EEG/EMG signals.

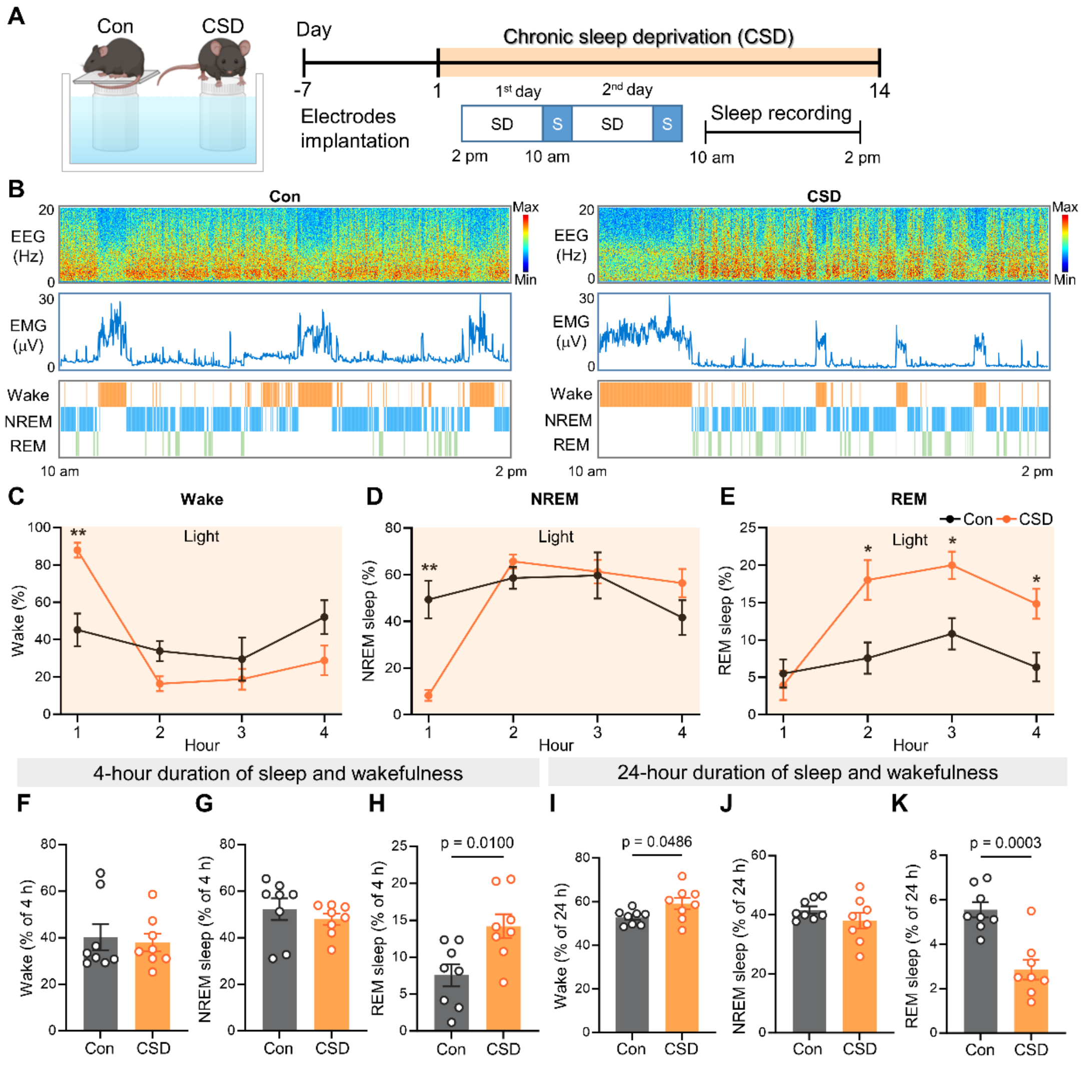


**Figure S2. The sleep-wake patterns during 4-h recovery period after sleep deprivation. (A)** Left, Schematic drawings of the sleep deprivation model. Right, Experimental design. Con, the control group; CSD, the chronic sleep deprivation group; SD, sleep deprivation; S, sleep. **(B)** Representative signals for EEG (upper), EMG (middle), and sleep architecture (bottom) during the 4-h recovery period in the homecage between the Control and CSD groups. EEG, electroencephalogram; EMG, electromyography; NREM, non-rapid eye movement; REM, rapid eye movement. **(C–E)** Representative graph of the hourly percentage of brain states from 10 am to 2 pm. **(F–H)** The percentage of each brain state over a 4-h recovery period. **(I–K)** The percentage of each brain state over a total 24-h duration including 20-h sleep deprivation period and 4-h sleep recovery period. Data are presented as mean ± SEM. *p < 0.05, **p < 0.01. n = 8 mice for each group.

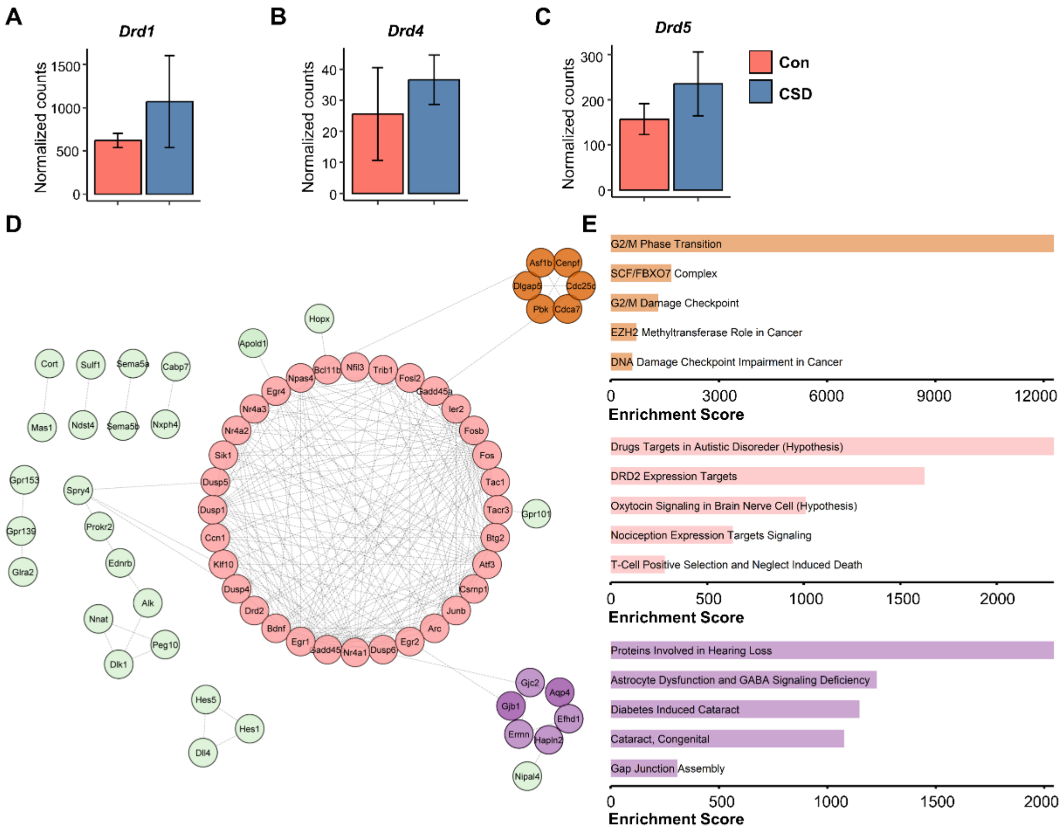


**Figure S3. The expression levels of other dopamine receptors and functional gene relationships following chronic sleep deprivation. (A–C)** Quantification of *Drd1*, *Drd4*, and *Drd5* gene expression between the Con and CSD groups. Con, the control group; CSD, the chronic sleep deprivation group. **(D)** Gene interaction network analysis of upregulated gene sets. **(E)** Gene sets were enriched for relevant diseases and annotated for functional analysis. n = 4 mice for each group.

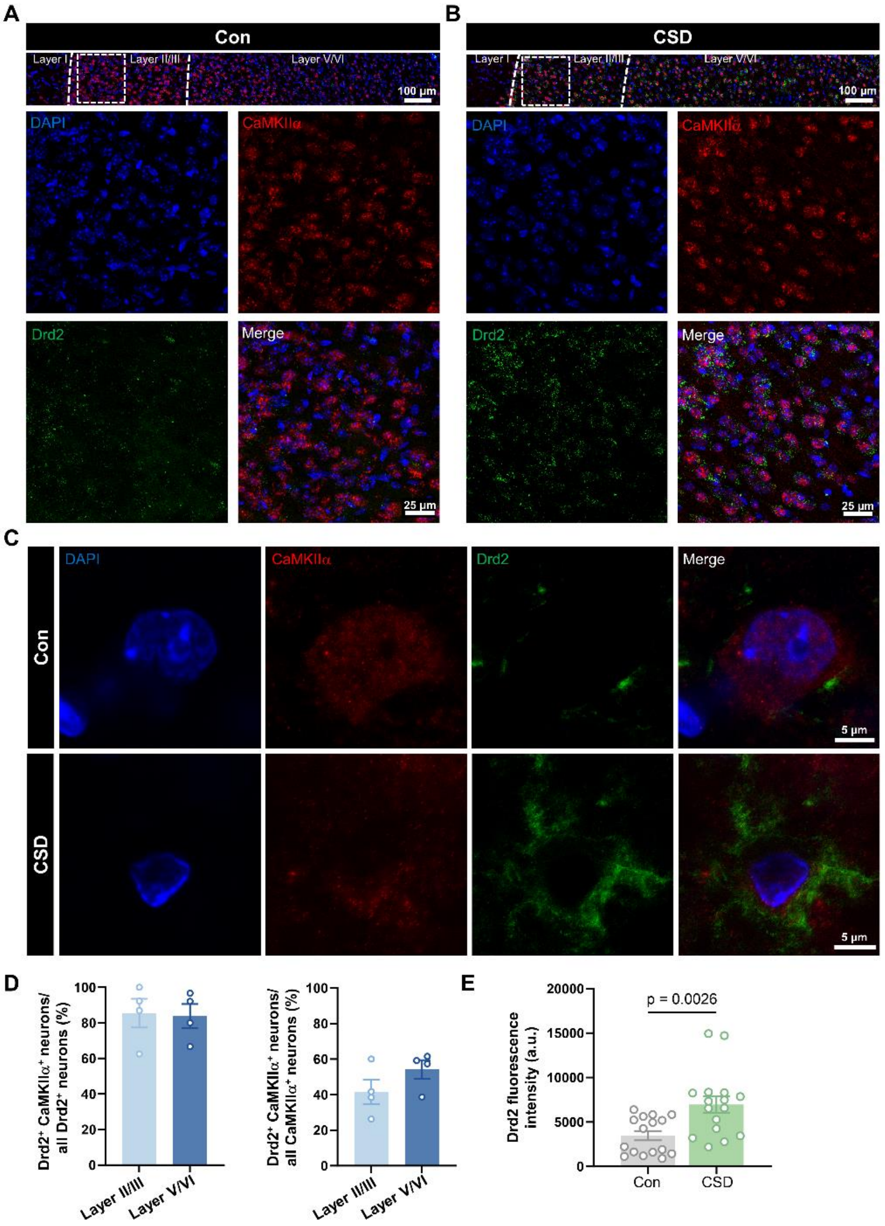


**Figure S4. Chronic sleep deprivation upregulated Drd2 protein expression in the mPFC. (A-B)** Top, Representative immunostaining images showing Drd2 protein expression in the mPFC of the Con and CSD groups. Bottom, Representative high-magnification image of the white-boxed area at the top of the main images. CaMKIIα (red), Drd2 (green), DAPI (blue). mPFC, medial prefrontal cortex; Con, the control group; CSD, chronic sleep deprivation. **(C)** STED images showing layer II/III of the mPFC of the Con and CSD groups. CaMKIIα (red), Drd2 (green), DAPI (blue). **(D)** Left, The percentage of Drd2$^{+}$ CaMKIIα$^{+}$ neurons among Drd2$^{+}$ neurons in different layers of the mPFC. Right, the percentage of Drd2$^{+}$ CaMKIIα$^{+}$ neurons among CaMKIIα$^{+}$ neurons in different layers of the mPFC. n = 4 mice for each group. **(E)** The fluorescence intensity of Drd2 of both groups. n = 16 from 4 mice for each group.

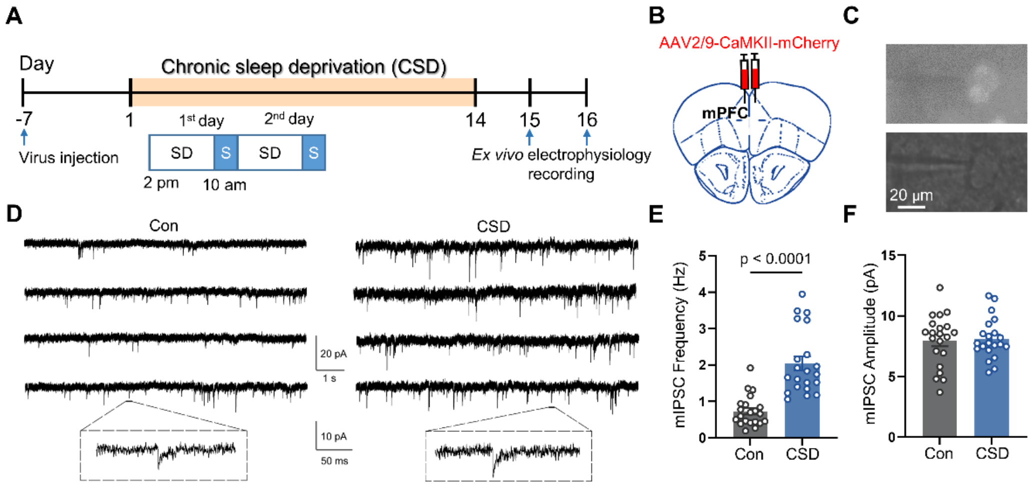


**Figure S5. Chronic sleep deprivation increased GABAergic transmission in layer II/III of the mPFC.** **(A)** Experimental design. SD, sleep deprivation; S, sleep. **(B)** Schematic of virus injection. mPFC, medial prefrontal cortex. **(C)** Fluorescence (top) and bright field (bottom) images showing whole-cell patch-clamp. **(D)** Sample traces showing mIPSCs of the Con and CSD groups. Con, the control group; CSD, the chronic sleep deprivation group. **(E)** Quantification of mIPSC frequency of two groups. **(F)** Quantification of mIPSC amplitude of two groups. mIPSC, miniature inhibitory postsynaptic current. n = 21 cells from 5 mice for each group.

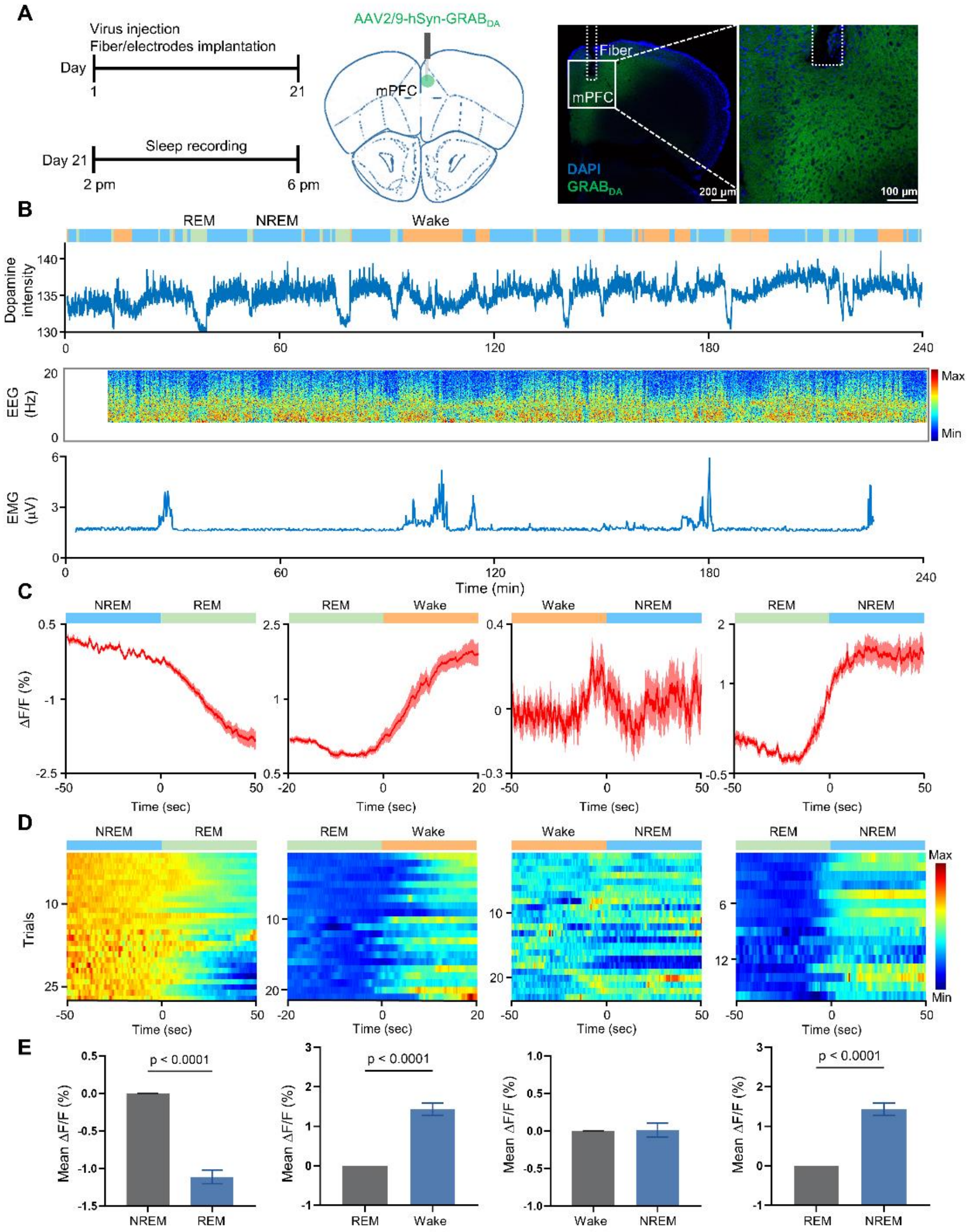


**Figure S6. Dopamine input to the mPFC decreased during REM sleep. (A)** Left, Experimental design. Middle, Fiber location and adeno-associated virus (AAV) injection. Right, Representative coronal brain tissue expressing $GRAB_{DA}$ (green). The white lines marked the position of an optical fiber. DA, dopamine; mPFC, medial prefrontal cortex. **(B)** Top, Representative sleep architecture, orange, blue, and green bars showed wakefulness, NREM sleep, and REM sleep, respectively. Middle, Representative traces of DA levels in the mPFC. Bottom, heatmap converted from EEG, and waveform of EMG. EEG, electroencephalogram; EMG, electromyography. **(C)** Temporal changes in DA levels at each brain state transition. Lines, mean across trials; shadings, ± SEM. NREM, non-rapid eye movement; REM, rapid eye movement. **(D)** Heatmaps showing DA level at each brain state transition.

Only state transitions where the preceding state lasted ≥10 s and the subsequent state lasted ≥10 s were extracted and converted for further processing. **(E)** Quantification of average DA level at each brain state transition. n = 3 mice, recorded for 4 h from 2 pm to 6 pm. One mouse was excluded due to a poor signal.

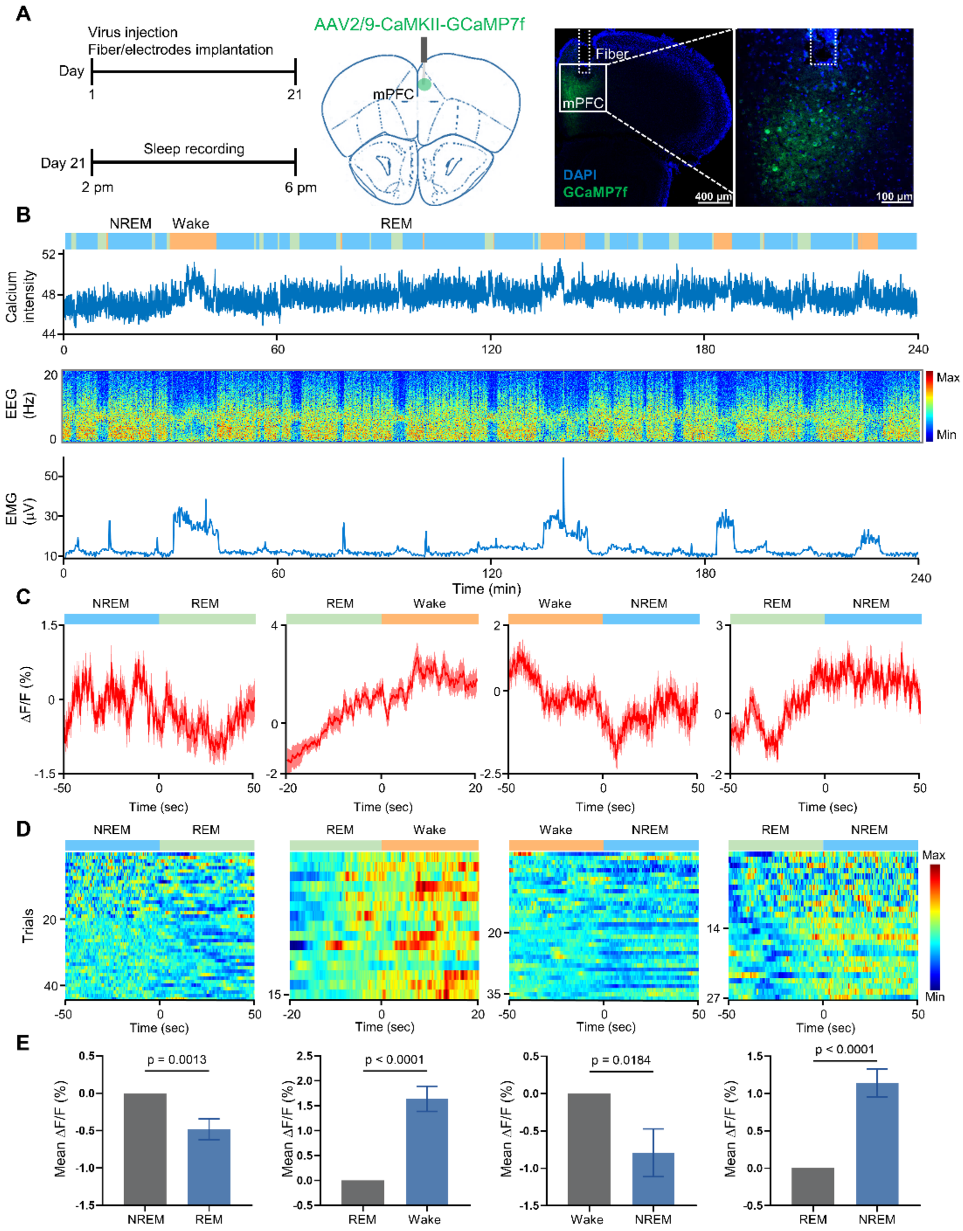


**Figure S7. Calcium activity of CaMKIIα⁺ neurons in the mPFC during sleep-wake cycle (A)** Left, Experimental design. Middle, Fiber location and virus injection. Right, Representative coronal brain section expressing GCaMP7f (green). The white lines marked the position of an optical fiber. mPFC, medial prefrontal cortex. **(B)** Top, Representative annotation of brain states, orange, blue, and green bars showed wakefulness, NREM sleep, and REM sleep, respectively. Middle, Representative traces calcium activity of CaMKIIα$^+$ neurons in the mPFC. Bottom, Heatmap converted from EEG, and waveform of EMG. EEG, electroencephalogram; EMG, electromyography; NREM, non-rapid eye movement; REM, rapid eye movement. **(C)** Temporal changes in calcium activity at each transition. Lines, mean across trials; shadings, ± SEM. **(D)** Heatmaps showing calcium activity at each transition.

Only state transitions where the preceding state lasted ≥ 10 s, and the subsequent state lasted ≥ 10 s were extracted and converted to data. **(E)** Quantification of average calcium activity at each brain state transition. n = 2 mice, recorded for 4 h from 2 pm to 6 pm. One mouse was excluded due to a missed fiber optic.

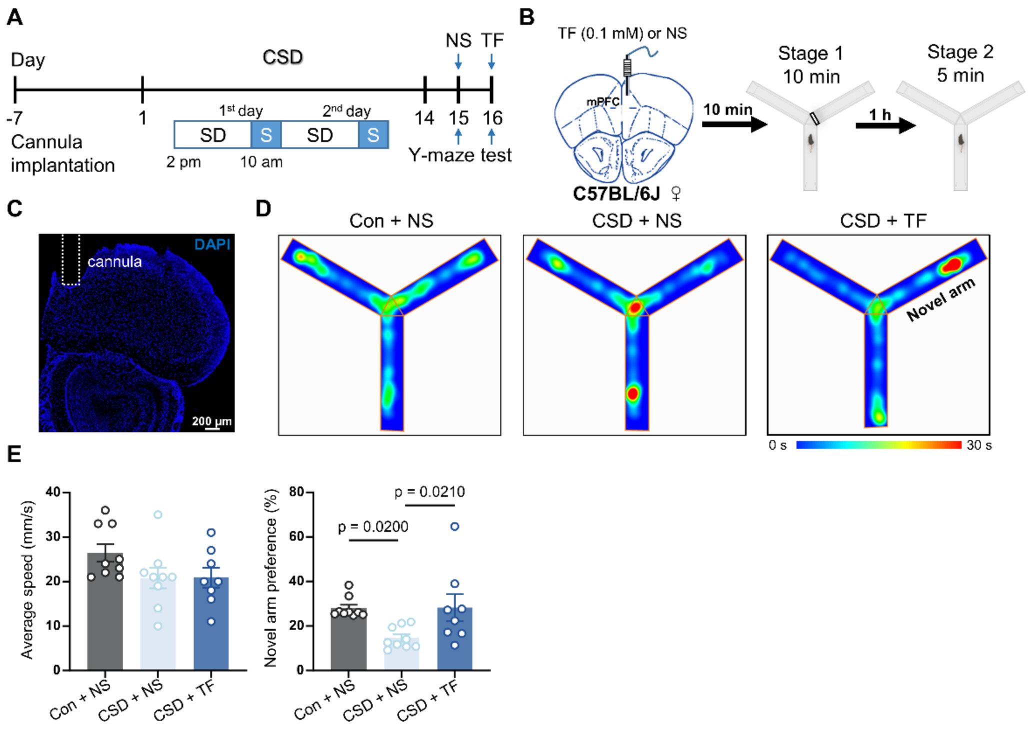


**Figure S8. Drd2 in the mPFC was involved in learning and memory in female mice. (A)** Experimental design. SD, sleep deprivation; S, sleep. **(B)** Schematic of the micro-infusion followed by the Y-maze test. The black line rectangle was represented as a barrier to close the novel arm during stage 1 of the Y-maze test. mPFC, medial prefrontal cortex; TF, Trifluoperazine 2HCl; NS, normal saline. **(C)** Representative image showing the position of a cannula in the mPFC. **(D)** Representative tracing heatmaps during the Y-maze test. **(E)** Quantification of average speed (left) and novel arm preference index (right). Con, the control group; CSD, the chronic sleep deprivation group. n = 9 mice for Con + NS and CSD + NS groups; n = 8 mice for CSD + TF group. One mouse was excluded due to an outlier.

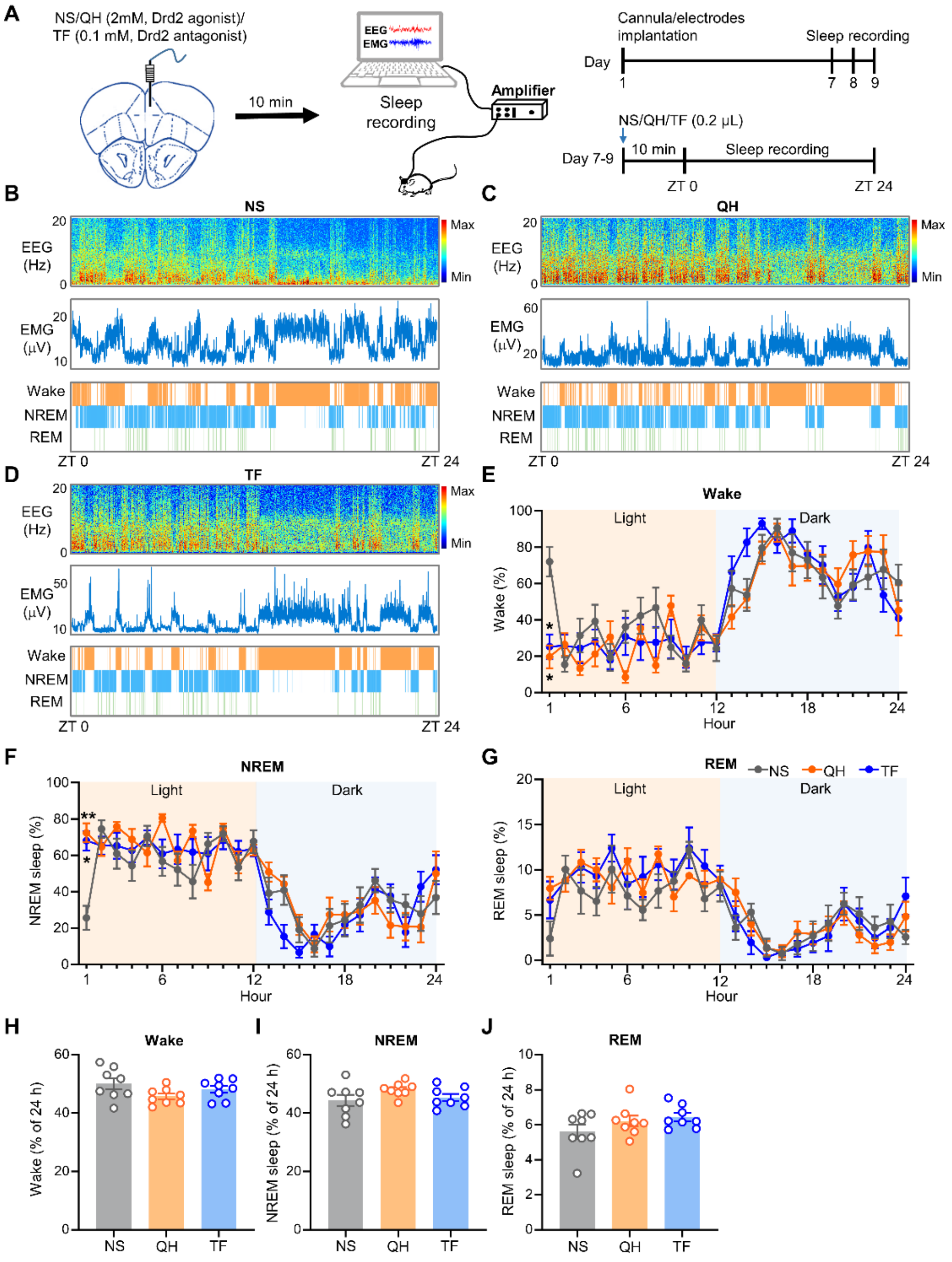


**Figure S9. Sleep and wakefulness states following Drd2 agonist or antagonist infusion. (A)** Schematic drawing of micro-infusion and sleep recording system (left) and experimental design (right). NS, normal saline; QH, Quinpirole hydrochloride; TF, Trifluoperazine 2HCl; ZT, zeitgeber time. **(B–D)** Representative signal for EEG (upper), EMG (middle), and sleep architecture (bottom) during the 24-hour free sleep from ZT 0 to ZT 24 of the normal saline (NS), Quinpirole hydrochloride (QH), and Trifluoperazine 2HCl (TF)-treated groups. EEG, electroencephalogram; EMG, electromyography; NREM, non-rapid eye movement; REM, rapid eye movement. **(E–G)** Representative graph of hourly percentage in sleep and

wakefulness states. *p < 0.05, **p < 0.01. **(H–J)** The average percentage of sleep and wakefulness states over a total duration of 24 h. Student's *t*-test was applied between the two groups. n = 8 mice for each group.

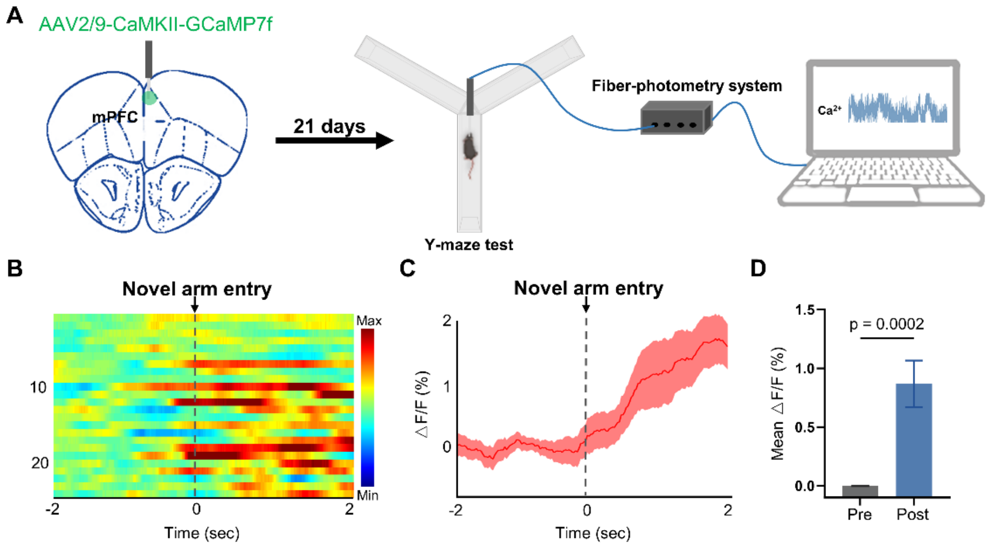


**Figure S10. Calcium activity of mPFC CaMKIIα⁺ neurons during the Y-maze test. (A)** Experimental design and schematic drawing of adeno-associated virus (AAV) injection, optical fiber location, and fiber-photometry system. mPFC, medial prefrontal cortex. **(B)** Heatmap showing activity of CaMKIIα⁺ neurons before and after moving into the novel arm during the Y-maze test. A total of 24 entries were recorded for 3 mice. **(C)** Change of CaMKIIα⁺ neurons activity before and after moving into the novel arm during the Y-maze test. The red line indicated the mean value and the shaded area indicated SEM. **(D)** Quantification of CaMKIIα⁺ neuronal activity before and after moving into the novel arm during the Y-maze test.

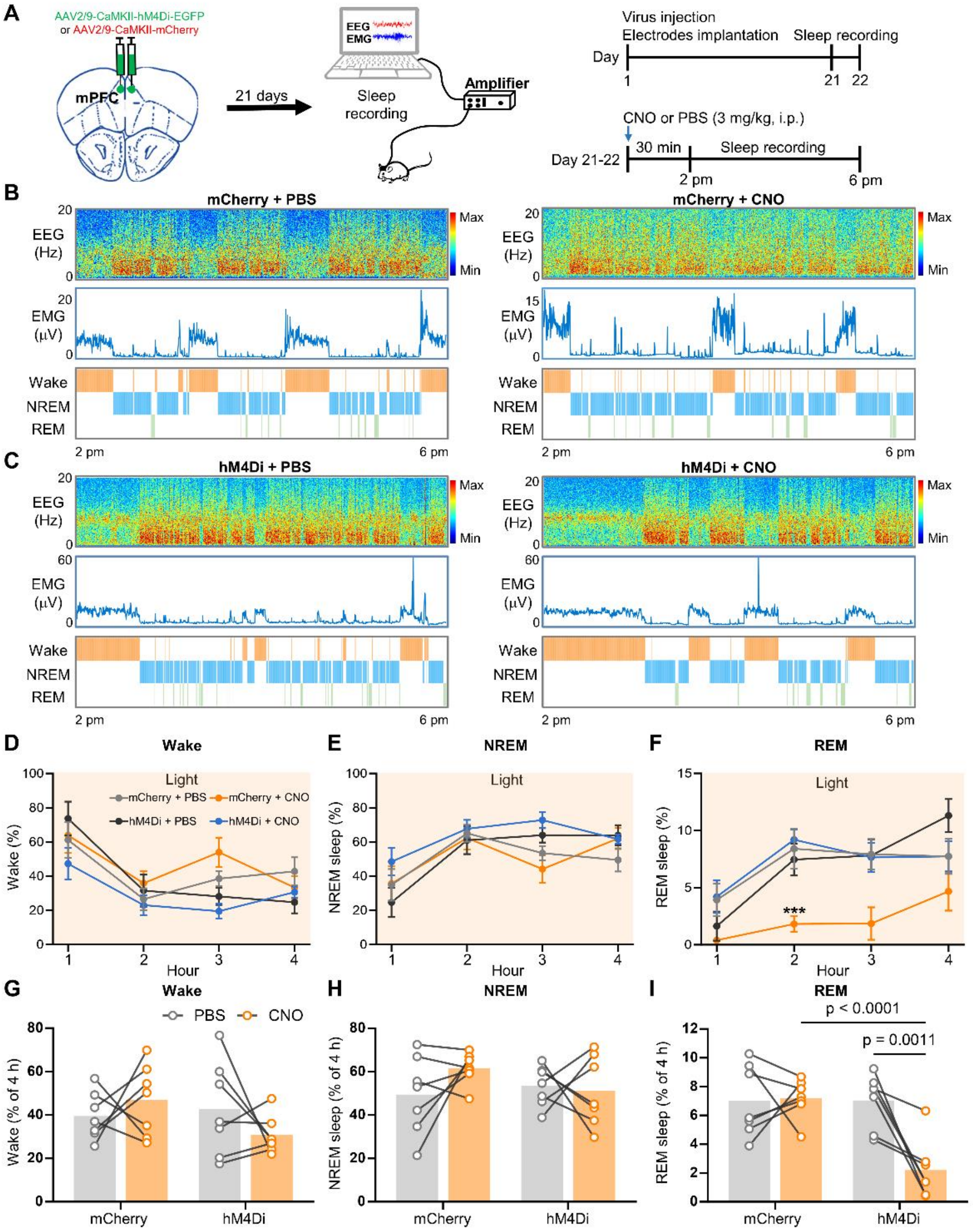

**Figure S11. Sleep and wakefulness states upon inhibition of mPFC CaMKIIα⁺ neurons. (A)** Schematic drawing of virus injection and sleep recording system (left), and experimental design (right). mPFC, medial prefrontal cortex; PBS, phosphate buffer saline; CNO, Clozapine N-oxide. **(B, C)** Representative signals for EEG (upper), EMG (middle), and sleep architecture (bottom) from 2 pm to 6 pm between the PBS and CNO-treated groups of mCherry- or hM4Di-expressing mice. EEG, electroencephalogram; EMG, electromyography; NREM, non-rapid eye movement; REM, rapid eye movement. **(D–F)** Plot of hourly percentage in sleep and wakefulness states. ***p = 0.001 (mCherry + CNO vs hM4Di + CNO). **(G–I)** The percentage of sleep and wakefulness states over 4-h recordings. n = 7 mice for each group.

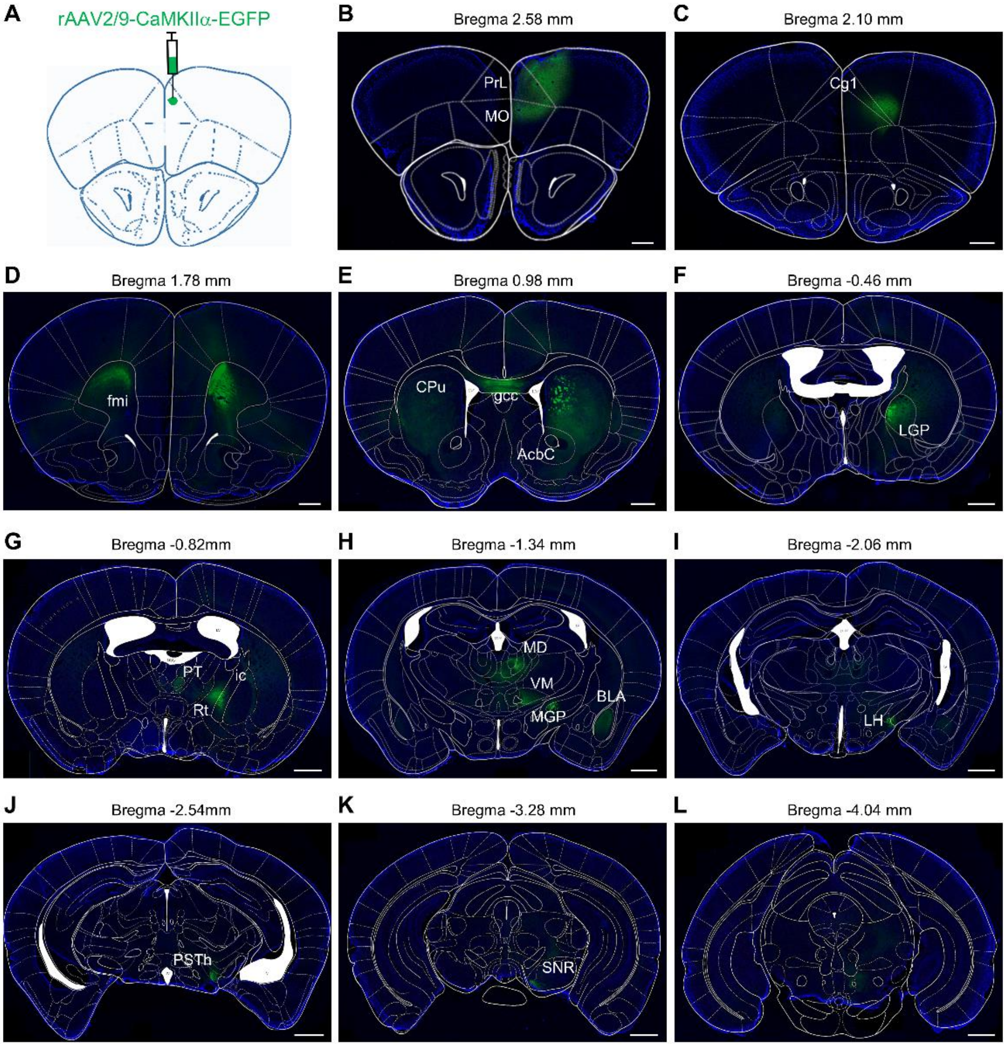


**Figure S12. Projections of CaMKIIα$^+$ neurons in the mPFC. (A)** Schematic of virus injection. **(B–L)** Representative images of the injection site in the mPFC and axonal terminals (green) in target structures. PrL, prelimbic cortex; MO, medial orbital cortex; Cg1, cingulate cortex area 1; fmi, forceps minor of the corpus callosum; CPu, caudate putamen (striatum); gcc, genu of the corpus callosum; AcbC, accumbens nucleus, core; LGP, lateral globus pallidus; PT, paratenial thalamic nucleus; Rt, reticular thalamic nucleus; ic, internal capsule; MD, mediodorsal thalamic nucleus; VM, ventromedial thalamic nucleus; MGP, medial globus pallidus (entopeduncular nucleus); BLA, basolateral amygdala; LH, hypothalamus; PSTh, parasubthalamic nucleus; SNR, substantia nigra, reticular part. Scale bars, 500 μm.
